# Femtosecond-to-Second Time-Resolved Spectroscopy Brings Unparalleled Insight Into the Life Cycle of the Versatile Manganese Photocatalyst [Mn_2_(CO)_10_]

**DOI:** 10.1021/jacs.5c16761

**Published:** 2026-04-09

**Authors:** Jonathan B. Eastwood, Conor D. Rankine, Thomas J. Burden, Abigail Frith, L. Anders Hammarback, Barbara Procacci, Daniel J. Shaw, Benjamin R. O’Donoghue, Ian P. Clark, Gabriel Karas, Partha Malakar, Gregory M. Greetham, Michael Towrie, Neil T. Hunt, Gerard P. McGlacken, Ian J. S. Fairlamb, Jason M. Lynam

**Affiliations:** † Department of Chemistry, 8748 University of York, York YO10 5DD, U.K.; ‡ School of Chemistry, Analytical & Biological Chemistry Research Facility, 8795 University College Cork, Cork T12 YN60, Ireland; § Central Laser Facility, Research Complex at Harwell, STFC, 97008 Rutherford Appleton Laboratory, Harwell Campus, Oxfordshire, Didcot OX11 0QX, U.K.

## Abstract

Visible-light-activated radical photoinitiators are pivotal in the efficient construction of complex molecular architectures and the precision synthesis of advanced polymeric materials. At the heart of their function lies the formation of reactive radical species that drive selective homolytic bond cleavage, but elucidating the fundamental mechanisms of these processes is notoriously difficult due to the fleeting nature of key intermediates. In this study, time-resolved infrared (TRIR) spectroscopy provides a powerful window into the complete reaction profile of the versatile photocatalyst [Mn_2_(CO)_10_], including the observation of [Mn­(O_2_)­(CO)_5_], which is a long-lived (ms) reservoir of the reactive 17-electron complex [Mn­(CO)_5_]. We give unprecedented structural and mechanistic insights concerning the formation of [Mn­(CO)_5_] in electronically and vibrationally excited states (fs–ps), its quenching by O_2_ (ns), regeneration of the ground-state catalyst, and C–I bond activation (ms). New avenues for the rational design of next-generation metal–metal photocatalysts are provided, as [Mn­(O_2_)­(CO)_5_] significantly extends the catalyst longevity.

## Graphical abstract

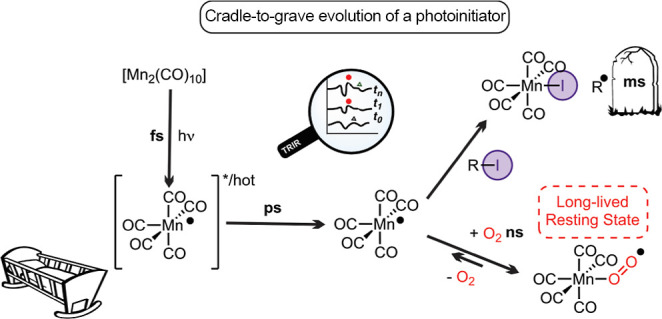

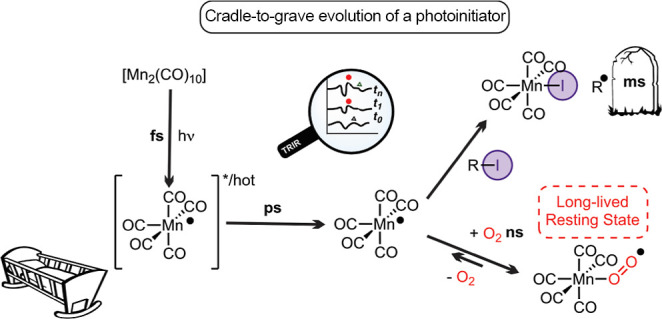

## Introduction

The use of light-driven reactions to promote the assembly of complex molecular frameworks is critical to modern chemical synthesis.^1–3^ The key factors underpinning these breakthroughs are inorganic and organic photosensitizers that absorb visible light, resulting in the subsequent generation of reactive, open-shell, intermediates.^4,5^ This can be achieved through a number of pathways; for example, the optically generated excited states of photosensitizers are simultaneously better oxidizing and reducing agents than their electronic ground states, thus enabling single-electron transfer reactions and access to doublet radicals. In addition, photosensitizers can promote energy-transfer processes which can sensitize reaction substrates to form triplet excited states, offering diverse reaction pathways when compared to their electronic ground states.^6^

An alternative method to access reactive radicals is through homolytic bond cleavage. Although this can be achieved directly through the use of either high-energy UV irradiation or thermal radical initiators, using a visible-light photoinitiator as a promoter has significant practical advantages.^7^ Transition-metal-based photoinitiators are attractive as they frequently have suitable absorption bands in the visible region of the spectrum which, on excitation, can generate reactive abstractor radicals through metal–metal or metal–ligand bond cleavage (Figure 1a). These abstractor radicals can then, in turn, activate a substrate to reveal a reactive organic radical that can undergo subsequent downstream chemistry and productive bond-forming processes.

**Figure 1:**
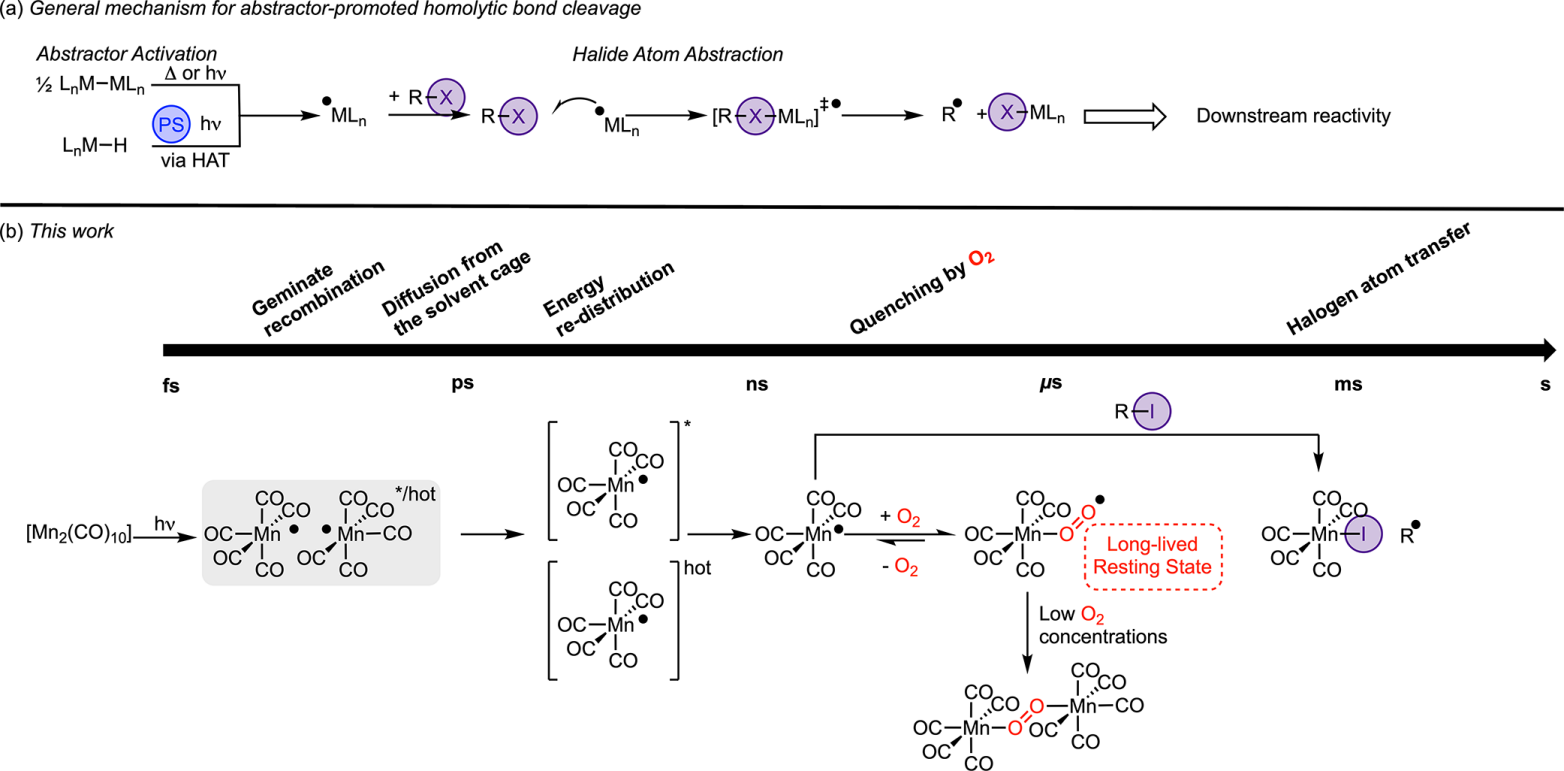
(a) General mechanistic steps for abstractor-initiated homolytic bond cleavage reactions using XAT as an exemplar (HAT = hydrogen-atom transfer, PC = photocatalyst). (b) Key findings from this work.

The dimeric manganese carbonyl complex [Mn_2_(CO)_10_] is a leading photoinitiator and has played a significant role in the development of new transformations for synthetic chemistry and as a versatile initiator for polymer synthesis.^8–11^ For example, light-activated [Mn_2_(CO)_10_] promotes (1) halogen-atom transfer (XAT) reactions by the abstraction of a halogen atom from a carbon–halogen bond to enable the assembly of diverse structural motifs;^8,12–25^ (2) radical polymerization processes initiated through carbon–halogen bond cleavage,^26,27^ (3) element–hydrogen bond activation to facilitate hydrosilylation and hydrogermylation,^14,28,29^ (4) RAFT (reversible addition-fragmentation chain-transfer) polymerization through C–S bond activation in dithiocarbonyls,^30^ and (5) N–Cl bond activation for the remote functionalization of amines.^31^

Mechanistically, it has been proposed that these reactions proceed via Mn–Mn bond cleavage to give 17-electron [Mn­(CO)_5_] which promotes homolytic bond cleavage in the desired substrate. This is consistent with previous time-resolved spectroscopic measurements, demonstrating that [Mn_2_(CO)_10_] may undergo two different light-driven processes. High-energy irradiation (λ < 400 nm) results primarily in CO dissociation to give [Mn_2_(CO)_9_],^32^ whereas the dominant pathway with lower-energy irradiation (λ > 400 nm) gives [Mn­(CO)_5_].^33–35^

In the case of carbon–halogen bond activation, blue-light-promoted radical reactions using [Mn_2_(CO)_10_] are therefore expected to give [MnX­(CO)_5_] (X = Cl, Br, I) through homolytic R–X bond cleavage and Mn–X bond formation. This process underpins the application of the photochemical functionalization of organic molecules through XAT reactions, where an organohalide is activated by carbon–halogen bond cleavage and the resulting carbon-based radical undergoes subsequent bond-forming reactions.^7^ In some cases, the initial halogen-atom transfer to manganese is thought to be followed by cyclization and then reabstraction of the halide from [MnX­(CO)_5_], a process proposed to be driven by the stronger C–X bond dissociation energies in the product when compared to the starting material.^12,13^

Developing a quantitative understanding of these Mn-promoted reactions is therefore important across a range of fields, especially as there is interest in the incorporation of alternative coligands into the coordination sphere of manganese, a process that enabled the development of new transformations for the hydrosulfonylation^36^ and hydrofluoroalkylation of alkenes,^37^ as well as CO_2_ reduction.^38^ A detailed mechanistic picture of these reactions would therefore enable the rational design of new metal–metal-based catalyst systems and transformations, complementing ubiquitous Ir-based photocatalyst systems. However, obtaining such information through direct observation of the key intermediates is extremely challenging, primarily due to the short lifetime of the radical species involved.

It was envisaged that the full life cycle of [Mn­(CO)_5_] could be observed under real catalytic conditions using time-resolved spectroscopy. Using an XAT reaction as an example system, this would include (1) capturing and understanding the ultrafast dynamics of its initial light-induced generation from [Mn_2_(CO)_10_], (2) providing insight into its subsequent interaction with the catalytic reaction medium, and (3) defining the ultimate role in halogen atom abstraction leading to substrate activation. Time-resolved infrared (TRIR) spectroscopy represents an ideal tool to obtain this mechanistic information as the vibrational modes of the manganese-bound carbonyl ligands are structure-sensitive probes of the metal coordination environment with high response factors. This allows the fate and dynamics of the complex to be readily determined.^39–52^ Experiments were designed in which an initial laser pump pulse (400 nm) would activate [Mn_2_(CO)_10_] and the subsequent formation and fate of [Mn­(CO)_5_] could be monitored through a subsequent probe pulse in the mid-IR region to observe the changes in the Mn–C≡O vibrational modes. Performing the experiments under the exact experimental conditions for XAT reactions would then enable the processes underpinning R–X bond activation to be directly observed, providing unique and quantitative insights into the nature of the mechanistic processes underpinning XAT reactions.

In addition, the Mn–Mn bond within [Mn_2_(CO)_10_] continues to be a significant computational chemistry challenge.^53–55^ As the 3*d*-valence orbitals on Mn have a limited radial extent,^56^ but there is a relatively long Mn–Mn bond distance (2.895(11)—2.923(3) Å ^53,57,58^ —single-crystal X-ray diffraction; 2.977(11) Å—gas-phase electron diffraction^59^), there is little orbital overlap between Mn atoms. It has been proposed that there is no covalent Mn–Mn bond and that a charge-shift bonding model better describes the ground-state electronic structure.^54,55^ Building on the hypothesis of using charge densities to quantify the bonding within [Mn_2_(CO)_10_],^53^ a recent quantum crystallography study reinforces the notion that there is little electron density accumulated between the Mn atoms in the dimer.^60^ Also, the recent syntheses of [FeMn­(CO)_10_]^+^ ^61^ and [Fe_2_(CO)_10_]^2+^,^62^ both of which are isoelectronic with [Mn_2_(CO)_10_], demonstrate the wider importance of obtaining a quantitative understanding of the structure, bonding, dynamics, and reactivity of this class of compound.

### Goals of This Study

The aim of this work was to investigate XAT reactions promoted by [Mn_2_(CO)_10_] using time-resolved multiple probe spectroscopy^63^ (TR^M^PS) on the LIFEtime spectrometer^64^ at the Rutherford Appleton Laboratory. By synchronizing the pump and probe laser pulses with different repetition rates, TR^M^PS was employed to record data with pump–probe delays ranging from 500 fs to 50 ms, allowing the fate of manganese over a wide temporal window to be observed. Furthermore, by integrating these measurements with time-resolved spectra using the rapid-scan IR technique, data covering a temporal range from 500 fs to minutes have been obtained, so the full life cycle of the XAT reaction can be observed (Figure 1b). The successful realization of this strategy is now reported, and unique insights into the catalytic processes underpinning the activity of [Mn_2_(CO)_10_] are reported. These include the observation of a Mn-based superoxide complex, which acts as a reservoir of [Mn­(CO)_5_], prolonging its lifetime. The outstanding spectroscopic resolution and dynamic range of the LIFEtime instrument have enabled hitherto undetected dynamics in the photochemistry of [Mn_2_(CO)_10_], related to the cleavage of the Mn–Mn bond and generation of the Mn­(CO)_5_ radical to be observed. These studies demonstrate how the nature of the Mn photocatalyst is profoundly affected by the reaction conditions employed and provide a guide for future synthetic efforts.

## Results and Discussion

### Femtosecond and Picosecond Dynamics of [Mn_2_(CO)_10_] and [Mn­(CO)_5_]

To investigate the mechanistic processes underpinning [Mn_2_(CO)_10_]-promoted XAT reactions, a strategy was envisaged in which the complexity of the experiment was systematically increased. In the first instance, the solution-phase behavior of [Mn_2_(CO)_10_] would be investigated and then experiments performed with the substrate added. This is a powerful method to observe directly key states in catalytic reactions as it enables the interactions in complex mixtures to be systematically deconvoluted.^39–41,45,46,52^

The initial studies focused on the photochemistry of [Mn_2_(CO)_10_] in different solvent media by using time-resolved spectroscopy. Visible,^32,65^ IR,^33^ or X-ray probes^34^ have been previously used to investigate the formation of [Mn­(CO)_5_], with the aim of delineating the full details of a complex reaction in operando. However, we recognized that the significantly greater spectroscopic resolution and dynamic range of the LIFEtime spectrometer, coupled with the wide temporal range of TR^M^PS, would allow for the complete evolution of the system to be obtained. This would provide a detailed cradle-to-grave map for XAT reactions and a global understanding of the factors controlling these reactions.

The photochemistry of [Mn_2_(CO)_10_] was, therefore, initially investigated in solvents with varying properties. Samples were activated by a short (150 fs) laser pulse (λ = 400 nm), and the subsequent changes in the vibrational spectra between ca. 1850 and 2200 cm^–1^ were used to rationalize the nature and dynamics of the resulting photoproducts. Data are presented as difference spectra, with negative peaks indicating species lost on irradiation and positive features corresponding to the newly formed photoproducts. The spectra collected in this manner for [Mn_2_(CO)_10_] in heptane are shown in Figure 2a. Three negative peaks were observed at 1983, 2014, and 2046 cm^–1^, corresponding to the *B*_2_‑, *E-,* and *B*_2_‑symmetric Mn–C≡O stretching modes of [Mn_2_(CO)_10_], respectively (black spectrum, Figure 2a),^66^ confirming that this molecule was lost on photolysis. At pump–probe delays between 1 ps and 1 ns, several important positive features were observed, providing information about the early time dynamics of the system. At 1 ps, the major positive band in these spectra was centered at ∼1994 cm^–1^ (Figure 2a, red circle) which, over the course of 20 ps (Figure 2b), decreased in intensity, coinciding with the buildup of the signal for two bands, ∼1987 and ∼1980 cm^–1^ (Figure 2a, green pentagon). These two new features were assigned to the *E-* and *A*_1_‑symmetric vibrational modes of *C*_4v_-symmetric^34,67^ [Mn­(CO)_5_], respectively. Although the identity of [Mn­(CO)_5_] was secured by comparison to the literature data,^67^ the species responsible for the peak at 1994 cm^–1^ has not been described previously.

**Figure 2:**
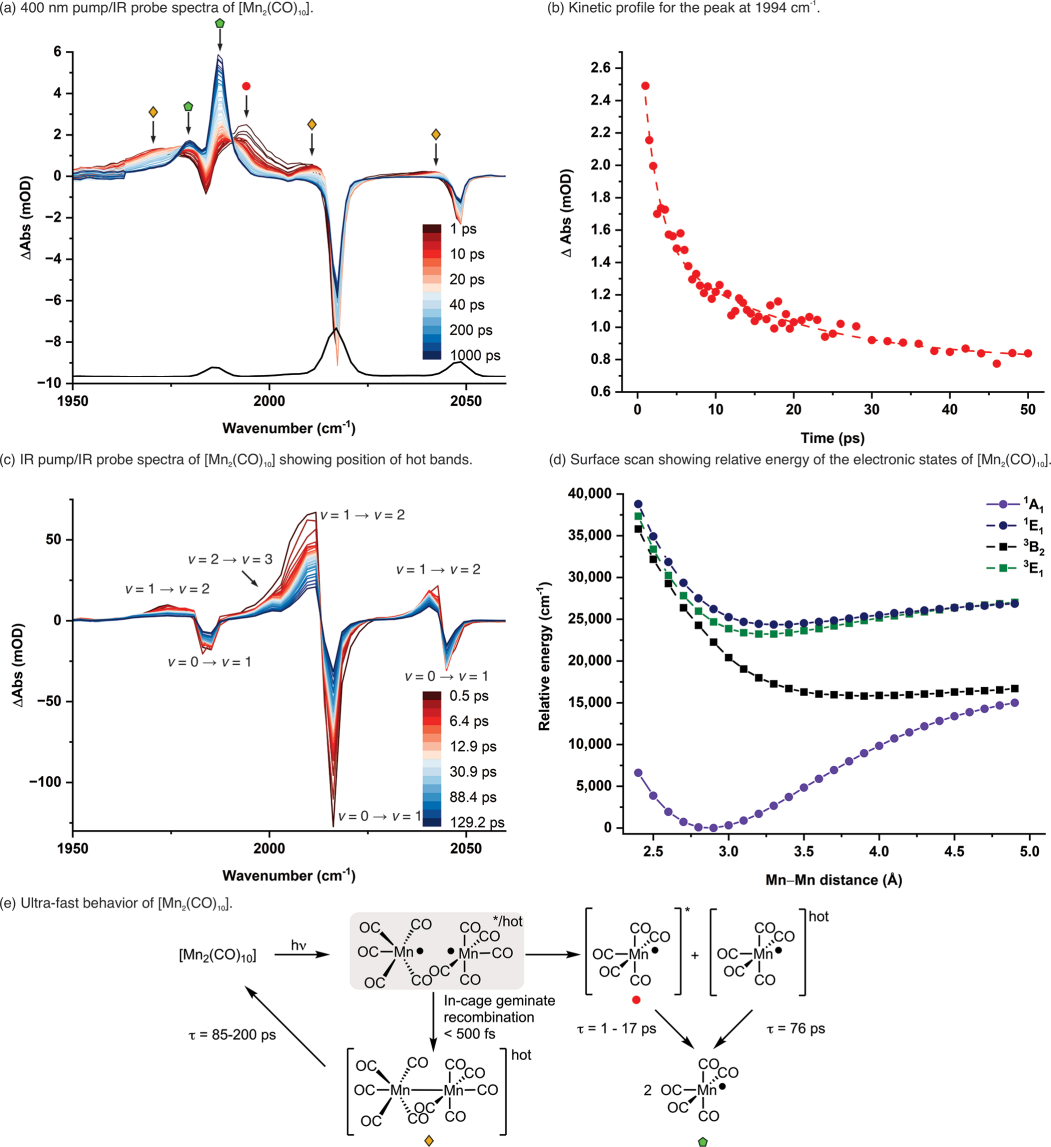
Ultrafast spectroscopy and calculations for the photolysis of [Mn_2_(CO)_10_]: (a) 400 nm pump/IR probe spectra of [Mn_2_(CO)_10_] in heptane solution, with pump–probe delays between 1 ps (dark red) and 1 ns (dark blue). The black spectrum is an FTIR spectrum of [Mn_2_(CO)_10_] in heptane. (b) Kinetic profile showing the temporal evolution of the peak at 1994 cm^–1^ between 1 and 50 ps. The dashed line is a fit to a biexponential function with τ = (1.9 ± 0.6) and (17.4 ± 9.1 ps) and *R* ^2^ = 0.974. An offset has been added to the fit to account for the overlap with the band due to [Mn­(CO)_5_]. (c) IR pump/IR probe spectrum of [Mn_2_(CO)_10_] in heptane solution with pump–probe delays between 0.15 ps (dark red) and 147 ps (dark blue), showing the band position for the vibrationally excited states. (d) Surface scan showing the relative energy of the lowest lying excited states of [Mn_2_(CO)_10_] as a function of Mn–Mn bond distance. Energies were determined at the CASPT2­(14,14)/ANO-RCC-VDZP level of theory. (e) Summary of the ultrafast behavior of [Mn_2_(CO)_10_]; the colored shapes assigned to the structures refer to the assignment given in (a).

The possibility of this band at 1994 cm^–1^ being due to vibrationally excited [Mn­(CO)_5_] ([Mn­(CO)_5_]^hot^) was excluded as the peak position is shifted to a higher frequency than the ν = 0 → ν = 1 transition in [Mn­(CO)_5_]; the anharmonic nature of the vibrational potential energy well entails that transitions between higher vibrational energy levels are at a sequentially lower energy. Furthermore, the possibility that the change in band position from 1994 cm^–1^ to 1987 and 1980 cm^–1^ was due to intramolecular vibration redistribution of [Mn­(CO)_5_] was also excluded, as this would again result in a shift in band position to a higher, rather than lower, energy.^68^

Several other assignments were considered. The possibility of the band being due to vibrationally excited [Mn_2_(CO)_10_] was investigated by using an IR pump/IR probe experiment using a spectrometer system in York. There, a 90 fs IR pump pulse excites the sample to a higher vibrational energy level, and the probe pulse then interrogates the nature of the ν = *n* → ν = *n* + 1 transitions. The resulting spectrum (Figure 2c) allowed for the identification of the peak positions of the ν = 1 → ν = 2 and ν = 2 → ν = 3 transitions for [Mn_2_(CO)_10_] (see Figure S19 for deconvolution).^69^ This revealed anharmonicity values of 10, 6, and 4 cm^–1^ for the *B*_2_, *E,* and *B*_2_ fundamental modes of [Mn_2_(CO)_10_], respectively.

Inspection of the 400 nm pump/IR probe data revealed that these spectroscopic features were also present (Figure 2a, yellow diamonds), confirming that [Mn_2_(CO)_10_]^hot^ had been formed in the experiment. The peaks for [Mn_2_(CO)_10_]^hot^ arise from the fact that the energy of the excitation photon is greater than the Mn–Mn bond dissociation energy, and therefore any [Mn_2_(CO)_10_] reformed by the geminate recombination of [Mn­(CO)_5_] will be in a higher vibrational energy level.^70^ These peaks decreased in intensity over ca. 200 ps with a commensurate recovery of the ground-state bleach bands: an observation again consistent with the intermolecular vibrational energy transfer to the bath. In the 400 nm pump/IR probe spectra, the lifetime for the recovery of the *E*-symmetric bleach band was (111 ± 18) ps, whereas from the IR pump/IR probe spectra, it has been shown to be (165 ± 2) ps.^69^ It is proposed that on the sub-ns timescale, the recovery of [Mn_2_(CO)_10_] is solely due to vibrational relaxation and that the small discrepancy in lifetimes between the methods is due to different vibrational excited-state populations being generated between the two experiments.^71,72^ Therefore, geminate recombination of two [Mn­(CO)_5_] radicals to form [Mn_2_(CO)_10_]^hot^ is faster than the 500 fs resolution of this experiment, and no recombination of [Mn­(CO)_5_] occurs between 500 fs and 1 ns. Crucially, the band at 1994 cm^–1^ was not present in the IR pump/IR probe experiments and, therefore, cannot be assigned to [Mn_2_(CO)_10_]^hot^.

The ground-state IR spectra of [Mn_2_(CO)_10_] in heptane were found to be temperature-independent over a range of 25–80 °C, excluding the possibility of a local heating effect altering the ground-state bleach position. Experiments were performed on different batches of [Mn_2_(CO)_10_] from different suppliers, and identical data were obtained in all cases, excluding the possibility that the peak at 1994 cm^–1^ was due to an impurity. In addition, the dynamics were unaffected by recording the experiments at different laser pump power levels. The possibility of a heteronuclear bond cleavage occurring first to give [Mn­(CO)_5_]^+^[Mn­(CO)_5_]^−^, prior to a single-electron transfer to give two [Mn­(CO)_5_] molecules (which may be viewed as the resonance extreme of the charge-shift bonding model),^55^ was also excluded as the peak at 1994 cm^–1^ did not correspond to the reported band positions for neither [Mn­(CO)_5_]^+^ ^73^ nor [Mn­(CO)_5_]^−^.^74,75^ In addition, this peak cannot be due to [Mn_2_(CO)_9_] (which is formed as a minor photoproduct at this excitation wavelength) as some of the characteristic peaks for this species (bands expected at 1975, 1997, 2016, 2039, and 2054 cm^–1^) were absent.^76,77^

The lifetime of the peak at 1994 cm^–1^ shows a small dependence on the nature of the solvent (see Supporting Information), but the data did not show a relationship between this lifetime and solvent viscosity, which previous works by Tyler^78–82^ on [Mo­(η^5^-C_5_H_4_Me)­(CO)_3_] have shown, dictates the rate of radical release from a solvent cage. Hence, it was concluded that the peak at 1994 cm^–1^ was not due to two molecules of [Mn­(CO)_5_] held within a solvent cage.

A consideration of the energetic landscape following the photodissociation of [Mn_2_(CO)_10_] provides insight into the assignment of this species responsible for the band at 1994 cm^–1^. Working in common units of cm^–1^, the Mn–Mn bond dissociation energy is reported to be between 8000 and 14,550 cm^–1^,^55,83–89^ which means, after 25,000 cm^–1^ (ca. λ = 400 nm) excitation and Mn–Mn bond cleavage, there is an excess energy between 17,000 and 10,450 cm^–1^. Some of this is manifested in [Mn­(CO)_5_] being formed in the vibrational excited states. However, [Mn­(CO)_5_] exhibits a low-energy absorbance at (ca. 810 nm),^32^ so it must possess electronic excited states with energies <12,346 cm^–1^. Consequently, the peak at 1994 cm^–1^ is assigned to [Mn­(CO)]_5_ in an electronic excited state, [Mn­(CO)_5_]*. The related generation of a photoproduct in an electronic excited state has also been reported for the following S–H bond dissociation in thiophenols.^90–92^

Over the course of ca. 200 ps, the bands for solvated [Mn­(CO)_5_] were observed to sharpen and were fitted to a Gaussian line shape, and the full-width half-height line width plotted against time. This demonstrated a monoexponential function with a lifetime, τ, of 76 ps. This change in line shape was attributed to intermolecular vibrational energy transfer from the vibrationally excited [Mn­(CO)_5_] ([Mn­(CO)_5_]^hot^) to the solvent bath, commensurate with previous ps studies.^33^

To explore the behavior of the complex as the Mn–Mn bond was broken, the nature of the excited states of [Mn_2_(CO)_10_], and their dependence on the Mn–Mn bond distance, was explored (Figure 2e). To model the multireference nature of the Mn–Mn bond dissociation event, the energies of the states were calculated at the CASPT2­(14,14)/ANO-RCC-VDZP level of theory using an active space comprising the Mn–Mn σ-bonding and σ*antibonding orbitals, two 3d_δ_ orbitals, three 3d_π_ orbitals, and six π*CO orbitals (see Supporting Information for details). The calculations demonstrated that the ^1^ *A*_1_ ground state had a minimum energy at a Mn–Mn bond distance of ca. 2.9 Å, consistent with experimental data.^53,57–59^ The behavior of the four excited states was also explored. The lowest lying singlet excited state was found to be an optically bright ^1^ *E*_1_ state, with the σ*­(Mn–Mn) ← 3d_π2_ character. The corresponding triplet state, ^3^ *E*_1_, was found to be almost isoenergetic at all Mn–Mn bond distances which, when taken with their spin–orbit coupling constant (calculated to be between 99 and 106 cm^–1^, depending on the Mn–Mn distance), would imply that there is rapid intersystem crossing between the two *E*_1_ states. Importantly, both *E*_1_ states had a local minimum with an Mn–Mn bond distance of ca. 3.3 Å, slightly longer than the ground state but demonstrating that they are nondissociative with respect to the Mn–Mn reaction coordinate. In contrast, the lowest lying triplet state, ^3^ *B*_2_, which has σ*­(Mn–Mn) ← σ­(Mn–Mn) character, is dissociative along the Mn–Mn coordinate at all bond distances examined, which when populated would result in the generation of two discrete [Mn­(CO)_5_] molecules.

The CASPT2­(14,14)/ANO-RCC-VDZP calculations also gave insight into the nature of the proposed electronic excited state [Mn­(CO)_5_]*. Exploration of the first electronically excited (1^2^ *E*)-state potential energy surface revealed that the Franck–Condon point for [Mn­(CO)_5_] lies at 11,050 cm^–1^ relative to the ground-state (1^2^ *A*_1_) minimum-energy geometry. However, the 1^2^ *E* potential energy surface in the region of the *C*_4v_-symmetric Franck–Condon geometry descends extremely steeply toward the 1^2^ *B*_1_/1^2^ *A*_1_ crossing seam, characterized by *C*_2v_ or *D*_3h_ symmetry (Figure S25). This situation is reminiscent of the states formed in the photodissociation of a CO ligand from Cr­(CO)_6_,^93^ in that the *D*_3h_ geometry is Jahn–Teller-unstable and sits at a conical intersection. The pathway between the *C*_4v_-symmetric 1^2^ *A*_1_ minimum-energy geometry and 1^2^ *B*_1_/1^2^ *A*_1_ crossing seam on the 1^2^ *A*_1_ potential energy surface is very shallow: the 1^2^ *B*_1_/1^2^ *A*_1_ minimum-energy crossing point is low-lying, being located at only 3910 cm^–1^ with respect to the 1^2^ *A*_1_ minimum-energy geometry.

It is therefore proposed that, following Mn–Mn bond cleavage, the excess internal energy (between 17,000 and 10,450 cm^–1^) in the [Mn­(CO)_5_] fragments is sufficient for the complex to explore both the ground (1^2^ *A*_1_) and low-lying excited-state (1^2^ *E*) potential energy surfaces via a pseudorotation-type mechanism that facilitates interconversion between the *C*_4v_ and *C*_2v_/*D*_3h_ symmetries and, consequently, the 1^2^ *A*_1_ and 1^2^ *B*_1_ electronic states. When this excess energy is dissipated, this is no longer possible, hence the disappearance of the peak at 1994 cm^–1^. At the CASSCF/ANO-RCC-VDZP level, the *C*_4v_ ground state of [Mn­(CO)_5_] is predicted to have MnC≡O bands at 2250 (*E*) and 2222 (*A*_1_) cm^–1^, whereas the corresponding stretches for the *C*_2v_ state in proximity to the 1^2^ *B*_1_/1^2^A_1_ crossing seam are at 2282 (*A*_1_), 2222 (*B*_2_), and 2197 (*B*_1_) cm^–1^. This indicates that the spectrum of the *C*_2v_/*D*_3h_-symmetric structure(s) should exhibit a peak at higher energy, consistent with the experimental observation of a band at 1994 cm^–1^.

A summary of all the ultrafast behaviors of light-activated [Mn_2_(CO)_10_] is shown in Figure 2e. The early time dynamics of [Mn­(CO)_5_] is now fully established.

### Nanosecond and Microsecond Observations of the Fate of [Mn­(CO)_5_]

Our next stage was to observe the key states involved in a Mn-promoted XAT reaction, and the radical cyclization reaction shown in Figure 3a was selected.^23^ In this reaction, it is proposed that the iodide abstraction from **1** by [Mn­(CO)_5_] results in the formation of [MnI­(CO)_5_] and an alkyne radical, **2**, which then undergoes cyclization to give **3** (Figure 3a). In the initial publication, it was proposed that a rebound reaction occurs between **3** and [MnI­(CO)_5_] to give **4**, regenerating [Mn­(CO)_5_].^23^ Interestingly, and of key importance to the findings in this work, the reaction functioned effectively under an air atmosphere with a yield similar to that obtained for a reaction under an N_2_ atmosphere.

**Figure 3:**
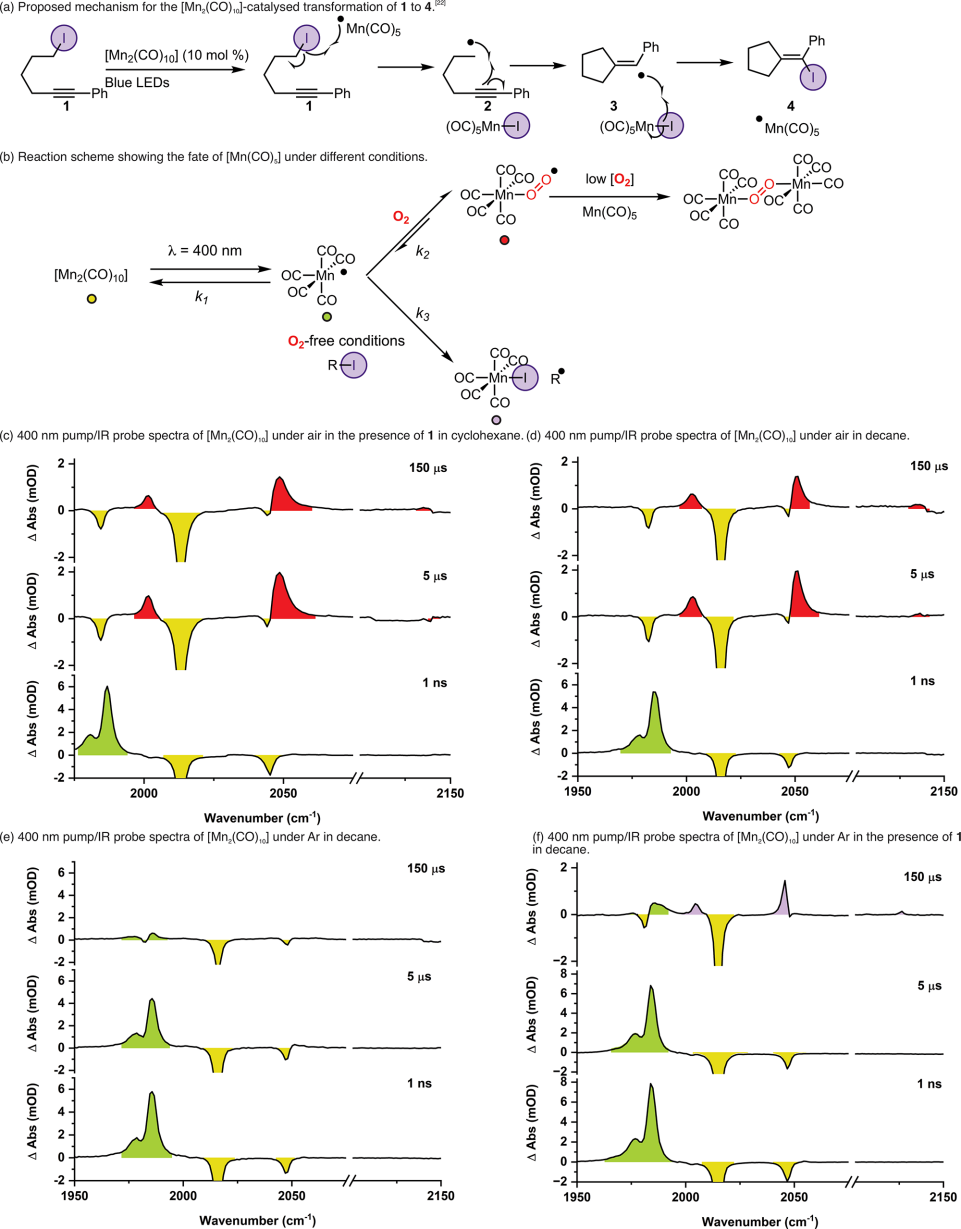
Spectroscopic and computational data for the interaction between light-generated [Mn­(CO)_5_] and O_2_ on ns–μs timescale. (a) Reaction scheme showing the proposed mechanism for the [Mn_2_(CO)_10_]-catalyzed conversion of **1** to **4**. (b) Summary of the key mechanistic findings from this work; the colored markers assigned to the compounds are consistent with the shading of the transient absorbance bands in the subsequent spectra. (c) 400 nm pump/IR probe spectra for a cyclohexane solution of [Mn_2_(CO)_10_] and **1** under an air atmosphere. (d) 400 nm pump/IR probe spectra for a decane solution of [Mn_2_(CO)_10_] under an air atmosphere. (e) 400 nm pump/IR probe spectra for a decane solution of [Mn_2_(CO)_10_] under an Ar atmosphere. (f) 400 nm pump/IR probe spectra for a decane solution of [Mn_2_(CO)_10_] and **1** under an Ar atmosphere. Bands for [Mn_2_(CO)_10_] are colored in yellow, [Mn­(CO)_5_] in green, [Mn­(O_2_)­(CO)_5_] in red, and [MnI­(CO)_5_] in purple.

The interaction of light-generated [Mn­(CO)_5_] with excess **1** was explored. To mimic the experimental synthetic conditions, cyclohexane was used as a solvent, and the experiments were performed under air.^23^ In some experiments, 1,4-diiodobutane was selected as a second, simpler reference substrate, and/or decane was used as a solvent. The early time (fs/ps) dynamics of the solutions containing **1** were unchanged from an experiment in cyclohexane alone. They were also essentially the same as in heptane (see earlier); however, over the course of ca. 1 μs, the band for [Mn­(CO)_5_] decreased in intensity to be replaced by three new bands at 2001, 2049, and 2136 cm^–1^, whose relative intensity and band positions are consistent with the *A*_1_^(1)^, *E,* and *A*_1_^(2)^ vibrational modes of a neutral *C*_4v_-symmetric complex of general formula [MnX­(CO)_5_] (Figure 3c).^93–95^ However, these peaks do not correspond to the band positions for authentic [MnI­(CO)_5_] (the expected product for iodide abstraction), and essentially identical observations were made when **1** was omitted from the experiment (Figure 3d), demonstrating that this new species was not formed by iodine atom abstraction. It was postulated that in the presence of air, [Mn­(CO)_5_] could undergo a nanosecond reaction with molecular oxygen to give [Mn­(O_2_)­(CO)_5_]. This species has previously been proposed on the basis of EPR studies^96–98^ and is speculated to be an intermediate in the recently reported bromination of alkenes^16^ and the functionalization of quinones.^99^

Consistent with the proposed formulation of [Mn­(O_2_)­(CO)_5_], an experiment performed under an O_2_ atmosphere^100^ resulted in the same spectroscopic features being present as under air, but the observed rate constant for their formation from [Mn­(CO)_5_] was a factor of 4 faster. Based on the mole fraction solubility for O_2_ in cyclohexane,^101^ the second-order rate constant for the reaction between [Mn­(CO)_5_] and O_2_ was calculated to be (3.29 ± 0.58) × 10^9^ mol^–1^ dm^3^ s^–1^. Furthermore, experiments performed in a deoxygenated cyclohexane or decane solution of [Mn_2_(CO)_10_] under argon demonstrated that only trace amounts of [Mn­(O_2_)­(CO)_5_] were formed, and the dominant process, occurring over ca. 150 μs, was the recombination of two molecules of [Mn­(CO)_5_] to reform [Mn_2_(CO)_10_] (Figure 3e). In these experiments, [Mn­(O_2_)­(CO)_5_] underwent a further reaction on a slower timescale to give a species with bands at 1997 and 2057 cm^–1^. Under these oxygen-limited conditions, it is proposed that the monoradical [Mn­(O_2_)­(CO)_5_] undergoes a further reaction with excess [Mn­(CO)_5_] to give [{Mn­(CO)_5_}_2_(μ-O_2_)].

A series of calculations were performed to understand the electronic structure of [Mn­(O_2_)­(CO)_5_]. A range of different methods were employed (see Supporting Information for details), but in all cases, the optimized geometry was a square-based pyramidal [Mn­(CO)_5_] group with an η^1^-bound O_2_ fragment (Figure 4). The three highest-energy α- and β-spin orbitals are shown in Figure 4, all of which may be viewed as having a π* character localized on the O_2_ ligand. Previous EPR studies on [Mn­(O_2_)­(CO)_5_] have shown that hyperfine coupling between the unpaired electron density and Mn nucleus is small,^96^ consistent with this computational picture. We suggest that [Mn­(O_2_)­(CO)_5_] is best treated as a Mn­(I) complex of a superoxide radical anion [O_2_^–.^], formed through the formal one-electron reduction of O_2_ by [Mn­(CO)_5_]. This picture is also supported by the similarity in the experimental and DFT-predicted (unscaled BP86/SV­(P)) IR spectra for [MnI­(CO)_5_] (experimental: 2004, 2045, and 2126 cm^–1^; predicted: 2035, 2057, and 2135 cm^–1^) and [Mn­(O_2_)­(CO)_5_] (experimental: 2001, 2049, and 2136 cm^–1^; predicted: 2029, 2042, 2047, 2063, and 2124 cm^–1^). The additional bands in the predicted spectrum of [Mn­(O_2_)­(CO)_5_] represent the deviation from the idealized *C*_4v_-symmetry in the calculated (static) structure.

**Figure 4:**
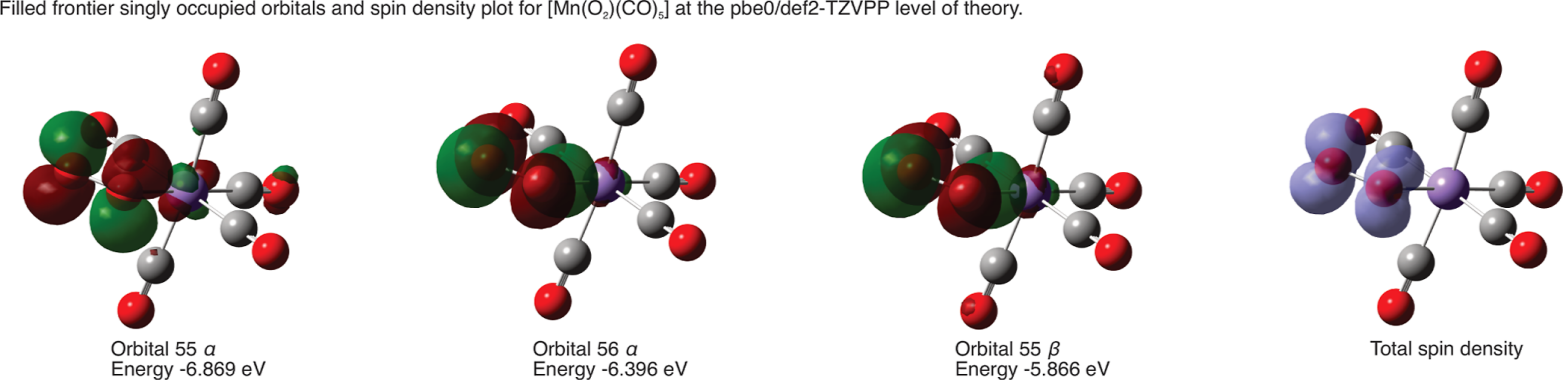
Two highest energy α-spin, the highest energy β-spin orbitals, and total spin density for [Mn­(O_2_)­(CO)_5_] at the UPBE0/def2-TZVPP level of theory.

Under an atmosphere of air (again mimicking the synthetic reaction conditions), little or no evidence for iodide abstraction from **1** was obtained on these timescales. However, photolysis of a solution of [Mn_2_(CO)_10_] and **1** in cyclohexane, which had been exhaustively deoxygenated by sparging with argon for >10 min, resulted in [Mn­(CO)_5_] loss and formation of [MnI­(CO)_5_] over 100 μs (Figure 3f), consistent with a model in which [Mn­(CO)_5_] has two competing fates, either iodide abstraction to give [MnI­(CO)_5_] or dimerization to reform [Mn_2_(CO)_10_]. Kinetic modeling (see Supporting Information for details) demonstrated that the rate constant of iodide abstraction from [**1**] by [Mn­(CO)_5_] was (1.75 ± 0.30) × 10^5^ mol^–1^ dm^3^ s^–1^. The dimerization of [Mn­(CO)_5_] to give [Mn_2_(CO)_10_] was shown to have a rate constant of (1.07 ± 0.10) × 10^9^ mol^–1^ dm^3^ s^–1^, the latter being consistent with previous reports.^102^

These experiments also demonstrated the extreme sensitivity of these systems to molecular oxygen. For example, in several instances, the repeated data acquisitions which are normally averaged as part of the experiment had a different composition, with the first run showing clean conversion to [MnI­(CO)_5_], but later runs forming both [MnI­(CO)_5_] and [Mn­(O_2_)­(CO)_5_], or [Mn­(O_2_)­(CO)_5_] alone, presumably due to trace air entering the experimental system. Furthermore, deliberate leaking of air into a sample that had solely formed [MnI­(CO)_5_] during its data acquisition and reacquiring the experiment resulted in the sole formation of [Mn­(O_2_)­(CO)_5_].

These data challenge the canonical view of XAT reactions as they unambiguously demonstrate that under air (and even trace amounts of O_2_), the primary kinetic fate of the [Mn­(CO)_5_] radical is not halide abstraction but formation of [Mn­(O_2_)­(CO)_5_]. A summary of these findings is shown in Figure 3b.

### Millisecond-to-Second Observation of the Key States in Photoinitiated C–X Bond Cleavage by [Mn­(CO)_5_]

The μs timescale data indicated that [Mn­(CO)_5_] reacts rapidly with O_2_ to give [Mn­(O_2_)­(CO)_5_]. Therefore, a key question remained: Under the synthetic reactions performed in air, how does C–I bond activation occur? To resolve the apparent dichotomy regarding the dominant formation of [Mn­(O_2_)­(CO)_5_] under the catalytic reaction conditions, the acquisition protocol for the TR^M^PS experiment was altered so that the pump pulse had a repetition rate of 20 Hz instead of 1 kHz while maintaining a 100 kHz probe repetition rate. This enabled measurements on light-activated [Mn_2_(CO)_10_] using TR^M^PS on timescales up to 50 ms, with data acquired every 10 μs. Cyclohexane solutions of [Mn_2_(CO)_10_] under air were investigated in both the absence (Figure 5a) and presence (Figure 5b) of 1,4-diiodobutane as a model substrate for iodide abstraction.

**Figure 5:**
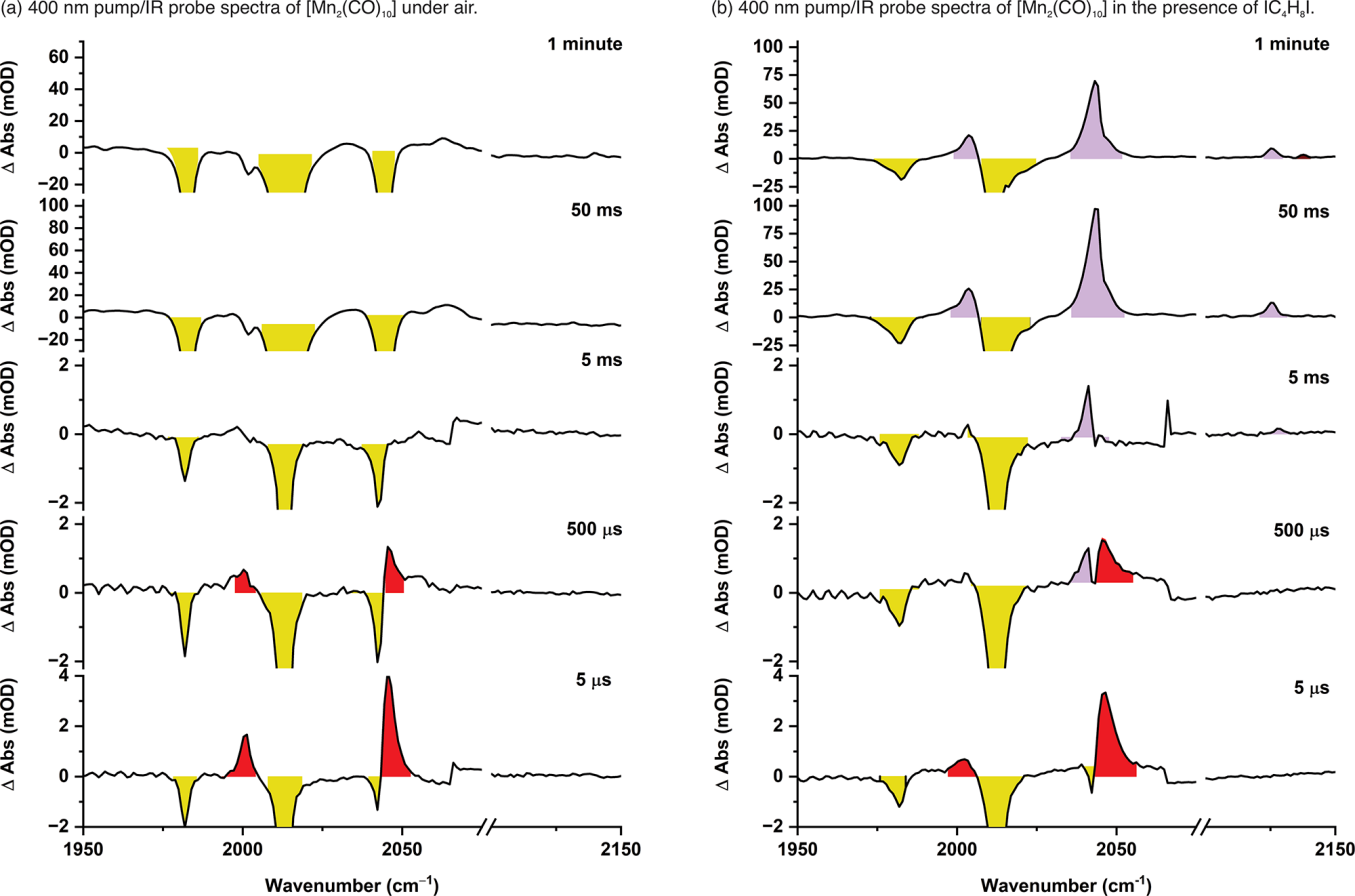
(a) 400 nm pump/IR probe spectra for a cyclohexane solution of [Mn_2_(CO)_10_] under an air atmosphere. (b) 400 nm pump/IR probe spectra for a cyclohexane solution of [Mn_2_(CO)_10_] and I­(CH_2_)_4_I under an air atmosphere. Bands for [Mn_2_(CO)_10_] are colored in yellow, [Mn­(O_2_)­(CO)_5_] in red, and [MnI­(CO)_5_] in purple. Spectra with a pump–probe delay of 5 μs, 500 μs, and 5 ms were acquired on the LIFEtime instrument with a probe repetition rate of 20 Hz. Spectra with a pump–probe delay of 50 ms and 1 min were acquired on a Bruker Vertex 80 instrument in Rapid Scan mode using 405 nm LED irradiation.

In the absence of substrates, the spectra showed the formation of [Mn­(O_2_)­(CO)_5_] as expected, but over the course of 2 ms, the peaks decreased in intensity. Some evidence for the negative bands for [Mn_2_(CO)_10_], also decreasing in intensity, was obtained, albeit over a slightly longer timescale. However, negative bands for [Mn_2_(CO)_10_] remained at longer times. When taken with the observation of small bands at 1998, 2030, and 2055 cm^–1^, this may represent the formation of a small amount of [Mn_2_(CO)_9_] by competitive CO loss, rather than Mn–Mn bond cleavage.^76,77,103,104^ Alternatively, it is possible that [Mn­(O_2_)­(CO)_5_] is unstable and may be undergoing decomposition.

Repeating the experiment in the presence of 1,4-diiodobutane showed the initial formation of [Mn­(O_2_)­(CO)_5_]. However, in this case, a smooth conversion to [MnI­(CO)_5_] was observed (Figure 5b). The loss of [Mn­(O_2_)­(CO)_5_] and the growth of [MnI­(CO)_5_] were successfully fitted to first-order kinetics with statistically identical rate constants. Repeating the experiment at double the concentration of 1,4-diiodobutane resulted in essentially identical data being obtained. Again, the observed rate constants in both experiments were statistically identical.

Kinetic modeling of these data (see Supporting Information) demonstrated that [Mn­(O_2_)­(CO)_5_] and [Mn­(CO)_5_] and O_2_ are in equilibrium. The loss of O_2_ from [Mn­(O_2_)­(CO)_5_] has a first-order rate constant of ca. 2 × 10^5^ s^–1^, and [Mn­(CO)_5_] thus generated may then undergo a competitive reaction with O_2_ to regenerate [Mn­(O_2_)­(CO)_5_], with 1,4-diiodobutane to produce [MnI­(CO)_5_], or dimerize to regenerate [Mn_2_(CO)_10_]. However, in the presence of 1,4-diiodobutane, the latter process is only a minor pathway.

Experiments were performed to study the behavior of the system on longer timescales (50 ms–min) using Rapid Scan FT-IR spectroscopy. At the earliest times investigated (50 ms), these experiments mirrored those from the TR^M^PS data on LIFEtime, demonstrating the selective formation of [MnI­(CO)_5_] when the experiment was performed in the presence of 1,4-diiodobutane and a spectrum with a low intensity in its absence.

These data therefore indicate that [Mn­(O_2_)­(CO)_5_] can undergo reactions with iodine-containing substrates to give [MnI­(CO)_5_]. A mechanistic picture in which there is an equilibrium between [Mn­(O_2_)­(CO)_5_] and [Mn­(CO)_5_]/molecular oxygen is consistent with these data. As no [Mn­(CO)_5_] is observed after ca. 0.5 ms, the spectroscopic data indicate that the equilibrium lies on the side of [Mn­(O_2_)­(CO)_5_]. However, the subsequent reactivity (reformation of [Mn_2_(CO)_10_] and iodide abstraction) illustrates that a small concentration of reactive [Mn­(CO)_5_] is present. Strictly speaking, an alternative pathway in which [Mn­(O_2_)­(CO)_5_] undergoes either a direct or indirect reaction with the alkyl iodide (i.e., not through the reversible formation of Mn­(CO)_5_) cannot be excluded on the basis of these data,^105^ in particular given that the superoxide complex undergoes an apparent decomposition in the absence of a substrate.

### Conclusions: A Global Mechanism for XAT by Light-Activated [Mn_2_(CO)_10_]

The results from this study have facilitated the deduction of the complete life cycle of a Mn carbonyl complex during an XAT reaction for the first time. This includes the fs–ps formation of the [Mn­(CO)_5_] radical and relaxation from its vibrationally and electronically excited states; its subsequent reaction with O_2_ to give [Mn­(O_2_)­(CO)_5_] on a ns–μs timescale, followed by a much slower (ms) halide abstraction reaction to give [MnI­(CO)_5_]. These are summarized in Figure 6, along with all of the rate constants determined for the uni- and bimolecular processes identified in this work. One of the major findings of this work is that, unless there is rigorous exclusion of molecular oxygen from a reaction mixture (i.e., use of a dry/glovebox), [Mn­(O_2_)­(CO)_5_] acts as the photocatalyst resting state, and this is important for the practicing synthetic chemist. Indeed, this may impart favorable outcomes on a given reaction, as [Mn­(CO)_5_] undergoes recombination to give [Mn_2_(CO)_10_] close to the diffusion-controlled limit. Instead, [Mn­(O_2_)­(CO)_5_] acts as a relatively long-lived (ms) store of [Mn­(CO)_5_] and is reminiscent of how N_2_ and THF coordination stabilizes the highly reactive “Cr­(CO)_5_” fragment through the formation of [Cr­(CO)_5_(N_2_)][Bibr ref106] and [Cr­(CO)_5_(THF)],^107^ respectively. This notion is reinforced in a recent report which demonstrated that the performance of light-activated [Mn_2_(CO)_10_] in a hydrosilylation reaction was found to be identically well under an atmosphere of either air, O_2_, or Ar.^29^

**Figure 6:**
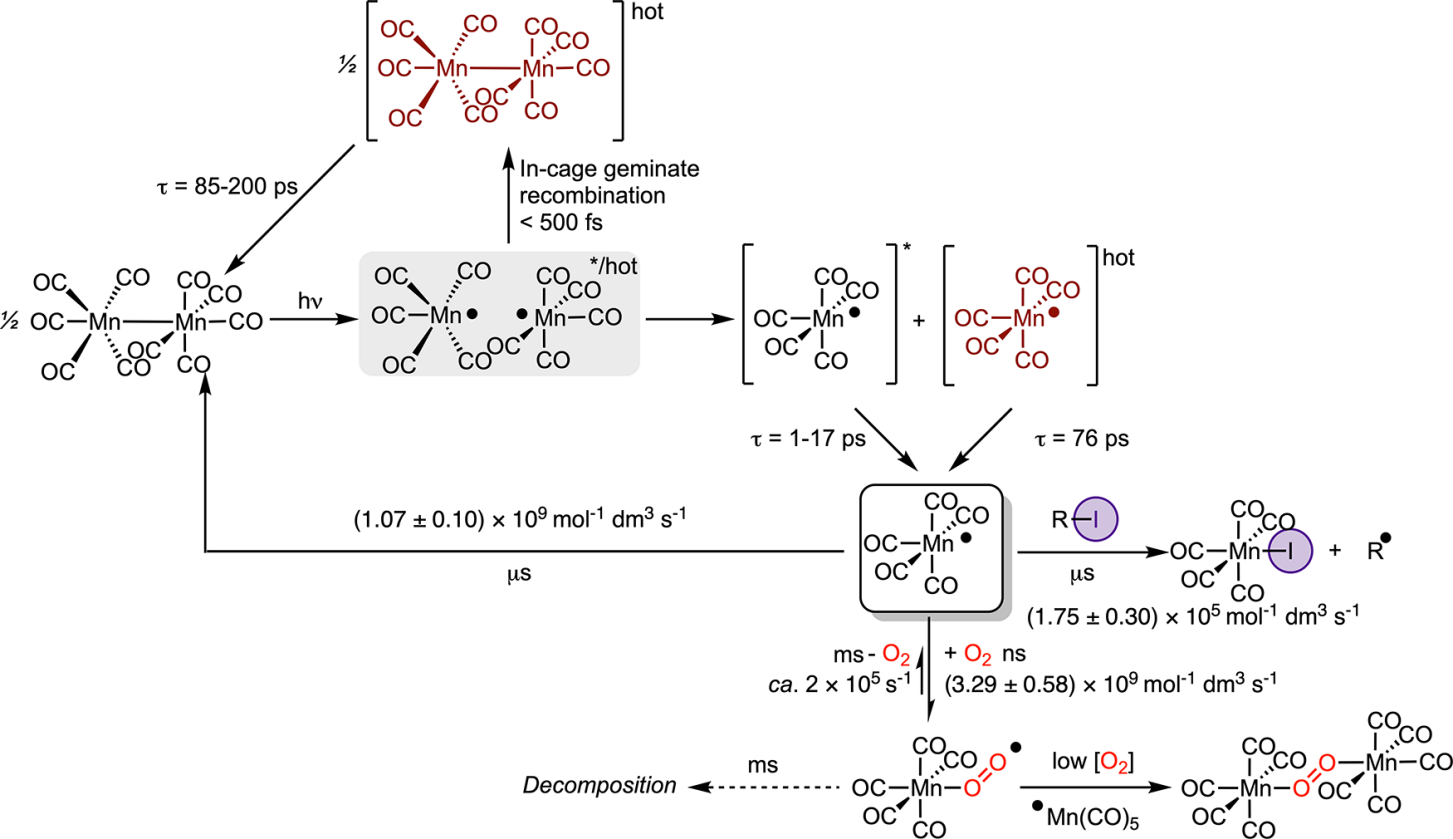
Global mechanism for R–I bond activation by [Mn­(CO)_5_]. The timescales given for a given process are representative of the concentration regimes used in this work.

In the case of [Mn­(O_2_)­(CO)_5_], it is proposed that the reactive [Mn­(CO)_5_] is leached into the solution at low concentrations, which limits unproductive dimerization but enables the desired halide abstraction reactions. It is therefore proposed that in some circumstances photoinitiated radical reactions may be actively promoted by molecular oxygen, and such conditions should be routinely interrogated in synthetic reaction screening protocols. The formation and decay of [Mn­(O_2_)­(CO)_5_] have implications for other photochemical reactions of [Mn_2_(CO)_10_] and other sources of [Mn­(CO)_5_]. Moreover, the global findings highlight the potential of metal–metal-based systems as potential photocatalysts in the future,^7^ as well as illustrating how the elucidation of the reaction mechanism by time-resolved spectroscopy can highlight the unexpected role of trace reaction components. Future studies will focus on understanding the role of the superoxide complexes in XAT reactions and will include an exploration of the role of the structure of the metalloradicals and the effects of O_2_ concentration on the reaction outcomes.

## Supplementary Material



## Notes

Details of the time-resolved spectroscopy experiment, data collection and processing, synthetic procedure and NMR spectrum of **1**, additional TRIR data, methodology for data collection under molecular O_2_, computational chemistry methods and further discussion (PDF)
The data supporting this research is openly available from the research data repository of the University of York at 10.15124/c0306cc5-6523-4889-a7c7-a6633d151526
