# Supplementary material for: Femtosecond-to-Second Time-Resolved Spectroscopy Brings Unparalleled Insight Into the Life Cycle of the Versatile Manganese Photocatalyst [Mn2(CO)10]
Source: J Am Chem Soc. 2026 Apr 9;148(15):15450–62. doi: 10.1021/jacs.5c16761 (PMC13107439; doi:10.1021/jacs.5c16761)
Supplement: Supplementary file 1 [file ja5c16761_si_001.pdf]

Supporting Information

Femtosecond To Second Time-Resolved Spectroscopy Brings Unparalleled Insight Into The Lifecycle Of The Versatile Manganese Photocatalyst $[Mn_2(CO)_{10}]$

Jonathan B. Eastwood,[a] Conor D. Rankine,[a] Thomas J. Burden,[a] Abigail Frith,[a,b] L. Anders Hammarback,[a] Barbara Procacci,[a] Daniel J. Shaw,[a] Benjamin R. O'Donoghue,[a,b] Ian P. Clark,[c] Gabriel Karas,[c] Partha Malakar,[c] Gregory M. Greetham,[c] Michael Towrie,[c] Neil T. Hunt,[a] Gerard P. McGlacken,[b] Ian J. S. Fairlamb*,[a] and Jason M. Lynam*,[a]

[a] Department of Chemistry, University of York, York, YO10 5DD, United Kingdom.

[b] School of Chemistry, Analytical & Biological Chemistry Facility, University College Cork, Cork, T12 YN60, Ireland.

[c] Central Laser Facility, Research Complex at Harwell, STFC Rutherford Appleton Laboratory, Harwell Campus, Didcot, Oxfordshire, OX11 0QX, United Kingdom.

Contents

1. General methods and procedures 3

2. Synthesis of iodoalkyne (6-iodohex-1-yn-1-yl)benzene, 1 7

3. Additional TRIR Spectra 8

4. Kinetic Modelling 17

4.1. Determination of k_1. 17

4.2. Determination of k_2 and k_3. 20

4.3. Determination of k_{-1}. 23

5. Procedure for Experiments Performed under Molecular O_2. 26

6. Computational Chemistry 29

6.1. Methodology 29

6.2. Evaluation of the structure and bonding in $[Mn(O_2)(CO)_5]$ 31

6.3. Predicted vibrational frequencies for key Mn complexes 33

6.4. Evaluation of the Excited State Manifold of $[Mn(CO)_5]$ 34

6.5. Collated Energies and xyz coordinates 35

7. References 48

1. General methods and procedures

Solvents and Reagents

Commercial chemicals were purchased from Acros Organics, Alpha Aesar, Apollo Scientific, Fisher Scientific, Fluorochem, Insight Biotechnology, Merck Life Science, Sigma-Aldrich, Strem Chemicals UK, or Tokyo Chemical Industry UK and were used without further purification unless otherwise stated. THF was was collected from a Pure Solv MD-7 solvent system and stored in oven-dried ampoules under an atmosphere of N_2.

Room-temperature (RT) typically refers to 21 ^{o}C, with an upper and lower limit of 16−23 ^{o}C recorded.

Chromatography

Thin-layer chromatography (TLC) was conducted using Merck aluminium-backed 5554 silica plates. Visualisation of spots was achieved *via* irradiation (254 nm), or sequential staining with potassium permanganate followed by heating. Flash column chromatography was carried out following the procedure reported by Still *et al.*,[1] using Fluorochem silica gel 60 (particle size 40−63 μm), with the solvent system stated in the specific procedure.

UV−Visible spectroscopy

UV−Visible spectra were recorded with a Jasco V−560 spectrometer using Quartz cuvettes.

Nuclear Magnetic Resonance Spectroscopy

Solution phase analysis was carried out on a Bruker AV500 spectrometer (500.13 MHz for ^{1}H) at 298 K. Spectra were processed in MestReNova software version 14.0.0-23239. In the ^{1}H spectra, coupling constants were quoted with ± 0.5 Hz. Chemical shifts are reported in ppm and referenced to the residual non-deuterated solvent.

Residual $CHCl_3$ in chloroform-d: ^{1}H: $CHCl_3$ 7.26 ppm.

^{1}H NMR peaks are reported to two decimal places.

Time-Resolved Multiple Probe Spectroscopy (TR^{M}PS)

TRIR measurements were carried out at the LIFEtime facility using the TR^{M}PS technique at the Central Laser Facility (Science and Technology Facility Council Rutherford Appleton Laboratories).[5,6] The experiments were driven by a 100 kHz repetition rate Yb:KGW amplifier (Pharos) as a pump source, producing 15 W, 260 fs pulses at 1030 nm. The laser output was used to drive a BBO-based 515 nm pumped optical parametric amplifier (OPA). The pump beam was collimated, travelled along a programable optical delay line (0-16 ns 1200 mm long double pass), then focused onto the sample. The probe beam sources were from a 100 kHz repetition rate YB:KGW amplifier(Pharos) producing 6W, 180 fs pulses at 1030 nm, driving two 3 W BBO/KTA based OPAs. The two Pharos sources shared a 80 MHz oscillator, allowing pump-probe delay steps of 12.5 ns. The probe beam was split to provide probe and reference pulses. The probe beams were collimated, synchronised by a fixed optical delay, and focused by a gold parabolic mirror onto the sample. The pump and two probe beams were overlapped on the sample using a 50 μm pinhole. The probe beams were measured by two separate 128-element detectors. To go beyond 12.5 ns, subsequent seed pulses can be selected from the 80 MHz oscillator.

Data were collected using two different pump repetition rates. For pump-probe delays ranging from 1 ps to 988.5 μs a pump repetition rate of 1 kHz was employed. Samples were prepared in an oven dried amberised Duran bottle. Approximately 5 mg of [$Mn_2(CO)_{10}$] was added and dissolved in 10 mL of anhydrous solvent from a newly opened sure-seal bottle. The system was then sparged (N_2, Ar, or air) for 10 minutes with solution pumping around the system. For the duration of the experiment, the Duran flask was sealed while under a positive pressure of sparge gas. The Duran bottle was connected *via* PTFE tubing to a Harrick cell with a spacer (100 μm unless stated otherwise), with solvent being pumped round the system using a peristaltic pump. During experiments, the Harrick cell was rastered in two dimensions, to prevent excitation of photoproducts. Following an experiment all solution was pumped from the system, 3 10 mL of new solvent pumped around to clean the kit, and dried using a positive pressure of N_2 for 10 minutes.

For experiments performed on a 5 µs – 50 ms timescale, a pump repetition rate of 20 Hz was employed, and samples were prepared in a static Harrick cell from a stock solution of 6 mg [$Mn_2(CO)_{10}$] in 10 ml cyclohexane. To avoid excess photochemical degradation during the experiments, samples were acquired with one negative and one positive pump-probe delay and averaged over five repeats. Samples were only rastered in a single direction.

Initially spectra were processed in ULTRA_VIEW_v2 where negative times were subtracted, and a polynomial second order baseline correction was applied, and data exported as a csv file. The resulting data were then analysed in OriginPro 2019b (64-bit) 9.6.5.169 (Academic) software. Where data sets were particularly noisy, early time points after the first TRMP were deleted and up to a 20-point average of data points applied. Kinetic fits were performed with appropriate ExpGro, ExpDec and ExpGroDec functions and values were quoted with in the format of XX ± XX indicating the 95% confidence limits of values obtained from exponential fits. The suitability of kinetic fits were then assessed using the built-in residual plots produced by OriginPro software.

IR Pump-IR Probe Experiments

IR Pump-IR Probe experiments were performed using a regeneratively amplified Ti:Sapphire laser system that produced mid-IR pulses via difference frequency mixing of the signal and idler beams generated by an optical parametric amplifier (OPA). The central frequency of the mid-IR pulses was selected to be resonant with the νCO modes of [$Mn_2(CO)_{10}$] (~ 2011 cm-1). The pulses had a bandwidth of 200 cm^{-1}, 100 fs pulse duration and were produced with a repetition rate of 1 kHz. The output of the OPA was split using a CaF_2 beamsplitter to give pump and probe pulse trains with intensity ratio (95:5). The two beams were overlapped spatially in the sample, and the pump-probe delay time was controlled via an optical delay line. After the sample, the probe beam was frequency dispersed using a spectrograph and detected using a 64 channel Mercury Cadmium Telluride (MCT, HdCdTe) array detector giving a probe frequency resolution of 4.5 – 5 cm^{-1}. For data acquisition, the pump beam was chopped at 500 Hz to allow collection of $pump_{on}$-$pump_{off}$ difference spectra. The delay time was scanned from –5 to 800 ps. Spectra were recorded obtained using magic angle

polarisation.

Rapid Scan experiments

Rapid scan experiments were performed on a Bruker Vertex V80 instrument.

A solution of 2 mg of [$Mn_2(CO)_{10}$] per 1 mL of solvent was injected into a Harrick cell with 200 µm spacers until the Harrick cell window was filled. A background spectrum was recorded prior to excitation, then controlled by the Brucker OPUS 8.5 software, a Thor Labs DC2200 Driver and Thor Labs 1.3 W 405 nm LED were triggered to give a 50 ms excitation pulse prior to measuring changes in the infrared spectrum. The spectra were recorded with a 2 cm^{-1} resolution, 1 sample scan, 1 background scan, between 1700 and 2200 cm^{-1} with an acquisition mode of double sided forward backward. Following data acquisition, preliminary processing was done in OPUS 8.5 software, converted to a .txt file, and further processing carried out in Origin Pro software.

Optics settings:

Source settings: MIR
Beam splitter: KBr
Optical Filter Setting: 6
Aperture setting: 1 mm
Accessory: Any
Measurement channel: Sample compartment
Background channel: Sample compartment
Detector setting: LN-MCT Photovoltaic Fast [Internal pos. 2]
Scanner velocity: 320 KHz
Sample Signal gain: x1
Sample preamp. gain: A
Background signal gain: x1
Background preamp. gain: A
Delay after device change: 0
Delay before measurement: 0

2. Synthesis of iodoalkyne (6-iodohex-1-yn-1-yl)benzene, 1

A Schlenk flask equipped with a magnetic stirrer bar was evacuated and backfilled with N_2 five times prior to addition of phenyl acetylene (0.65 mL, 6.0 mmol, 1.0 equiv.) and dry N_2-sparged THF (30 mL). The resulting solution was cooled down to -78 °C *via* an acetone-dry ice bath and *n*-BuLi (4 mL, 2.0 M) was added dropwise *via* an argon-purged syringe. The reaction mixture was stirred at -78 °C for 30 minutes and then warmed up to room temperature where 1,4-diiodobutane (1.2 mL, 9.0 mmol, 1.5 Equiv.) was added and the reaction mixture heated to 70 °C overnight. The reaction mixture was then cooled down to room temperature, quenched with a saturated solution of NH_4Cl and extracted with Et_2O (3 x 10 mL). The organic layers were combined, dried with $MgSO_4$, and concentrated in vacuo. The crude material was purified by flash column chromatography (Hexane) to deliver the product in 1.35 mg, 80 % yield, as a colourless liquid.

^{1}H NMR (500 MHz, $CDCl_3$) δ = 7.40-7.38 (m, 2H), 7.30-7.27 (m, 3H), 3.25 (t, J = 6.8 Hz, 2H), 2.46 (t, J = 6.8 Hz, 2H), 2.04-1.99 (m, 2H), 1.76-1.70 (m, 2H) ppm.

NMR Data. Data were in accordance with that reported in the literature.[1]

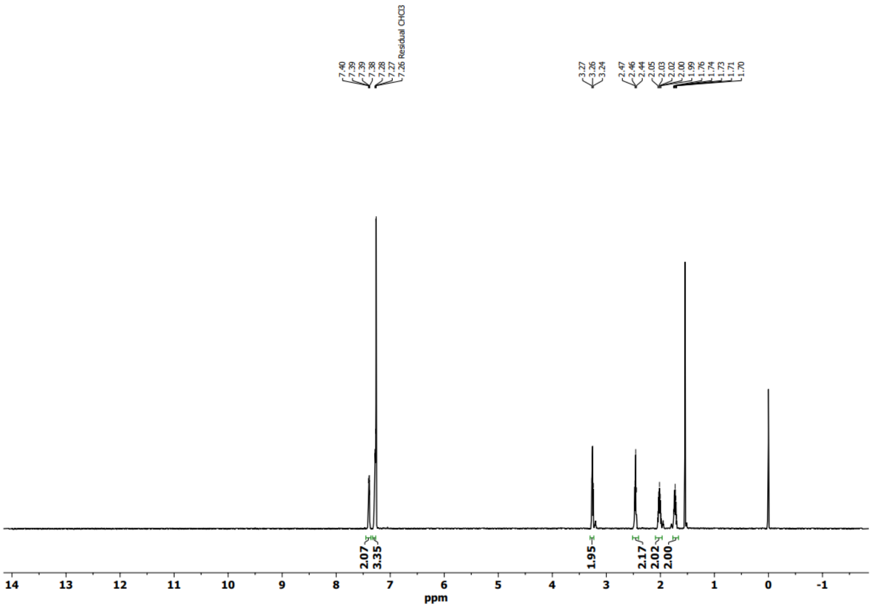

Figure S1. ^{1}H NMR spectrum of (6-iodohex-1-yn-1-yl)benzene in $CDCl_3$ recorded on a Brucker AV500 spectrometer.

3. Additional TRIR Spectra

All kinetic parameters from the fits below are collated in Table S1

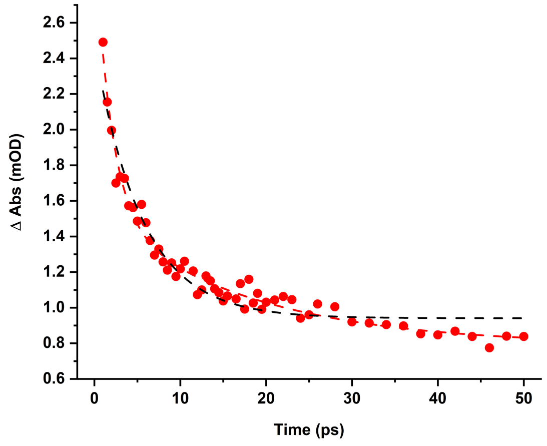

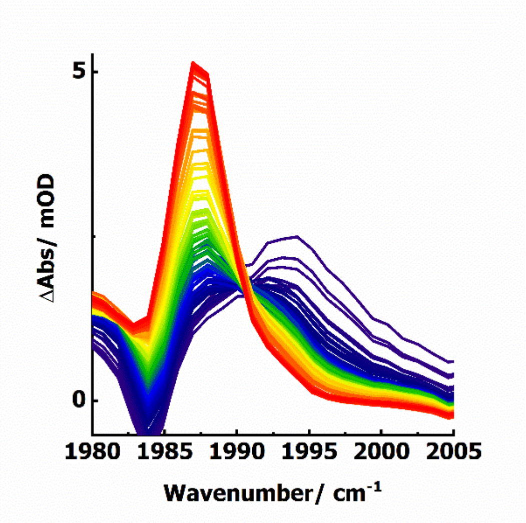

Figure S2. Approximately 5 mg of [$Mn_2(CO)_{10}$] per 10 mL of n-heptane solvent pumped with a 400 nm excitation wavelength following the general procedure for time-resolved multiple probe spectroscopy. Left: Kinetics for the loss of the initial photoproduct. The black dashed line is a fit to a monoexponential function, the red dashed line a fit to a biexponential function. Right: Spectra over the first 20 ps with purple, blue, green, yellow, orange, and red progressing from t = 0 ps to t = 20 ps.

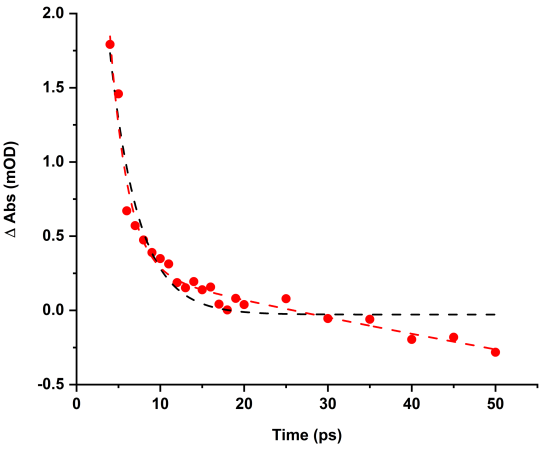

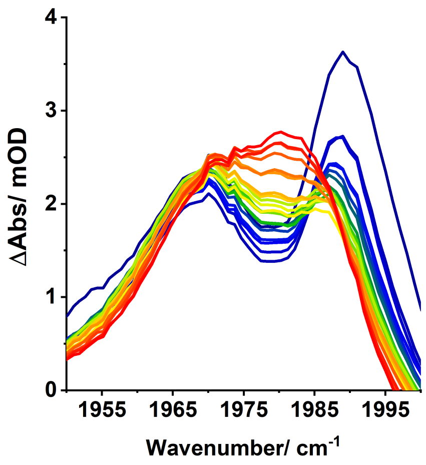

Figure S3. Approximately 5 mg of [$Mn_2(CO)_{10}$] per 10 mL of toluene solvent pumped with a 400 nm excitation wavelength following the general procedure for time-resolved multiple probe spectroscopy. Left: Kinetics for the loss of the initial photoproduct. The black dashed line is a fit to a monoexponential function, the red dashed line a fit to a biexponential function. Right: Spectra over the first 20 ps with purple, blue, green, yellow, orange, and red progressing from t = 0 ps to t = 20 ps.

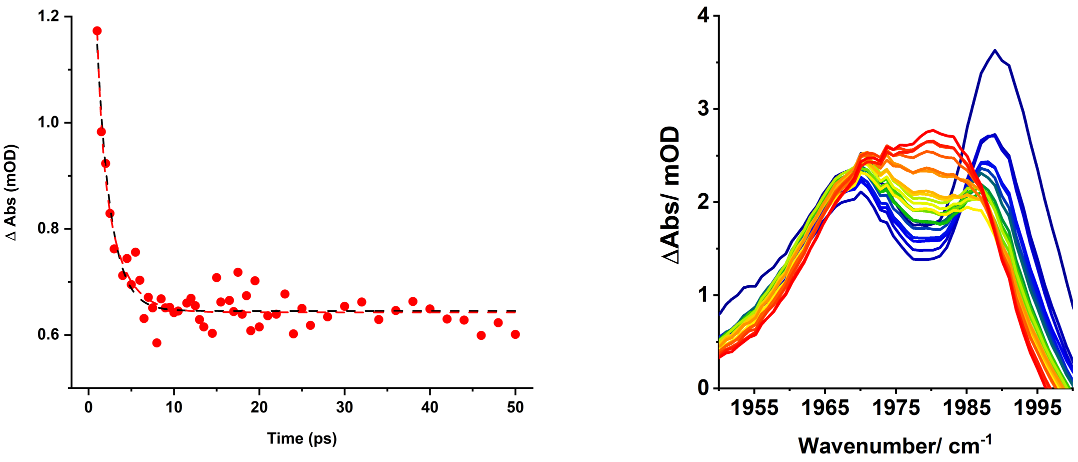

Figure S4. Approximately 5 mg of [$Mn_2(CO)_{10}$] per 10 mL of acetonitrile solvent pumped with a 400 nm excitation wavelength following the general procedure for time-resolved multiple probe spectroscopy. Left: Kinetics for the loss of the initial photoproduct. The black dashed line is a fit to a monoexponential function, the red dashed line a fit to a biexponential function. Right: Spectra over the first 20 ps with purple, blue, green, yellow, orange, and red progressing from t = 0 ps to t = 20 ps.

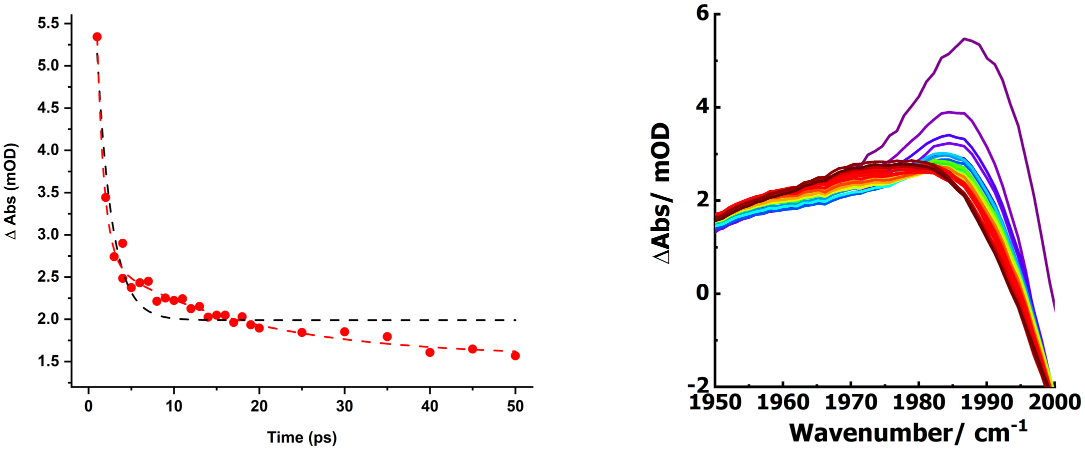

Figure S5. Approximately 5 mg of [$Mn_2(CO)_{10}$] per 10 mL of DMSO solvent pumped with a 400 nm excitation wavelength following the general procedure for time-resolved multiple probe spectroscopy. Left: Kinetics for the loss of the initial photoproduct. The black dashed line is a fit to a monoexponential function, the red dashed line a fit to a biexponential function. Right: Spectra over the first 20 ps with purple, blue, green, yellow, orange, and red progressing from t = 0 ps to t = 20 ps.

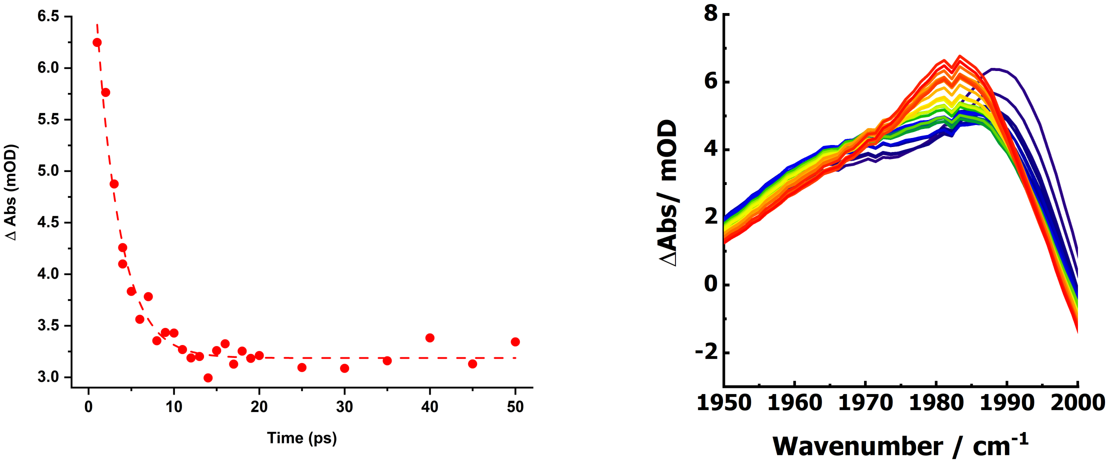

Figure S6. Approximately 5 mg of [$Mn_2(CO)_{10}$] per 10 mL of acetone solvent pumped with a 400 nm excitation wavelength following the general procedure for time-resolved multiple probe spectroscopy Left: Kinetics for the loss of the initial photoproduct. The black dashed line is a fit to a monoexponential function, the red dashed line a fit to a biexponential function. Right: Spectra over the first 20 ps with purple, blue, green, yellow, orange, and red progressing from t = 0 ps to t = 20 ps.

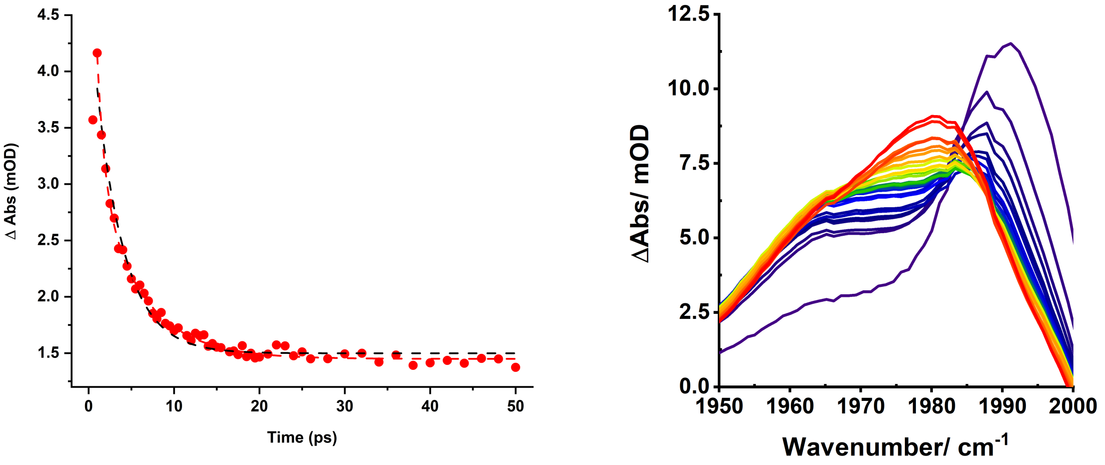

Figure S7. Approximately 5 mg of [$Mn_2(CO)_{10}$] per 10 mL of THF solvent pumped with a 400 nm excitation wavelength following the general procedure for time-resolved multiple probe spectroscopy Left: Kinetics for the loss of the initial photoproduct. The black dashed line is a fit to a monoexponential function, the red dashed line a fit to a biexponential function. Right: Spectra over the first 20 ps with purple, blue, green, yellow, orange, and red progressing from t = 0 ps to t = 20 ps.

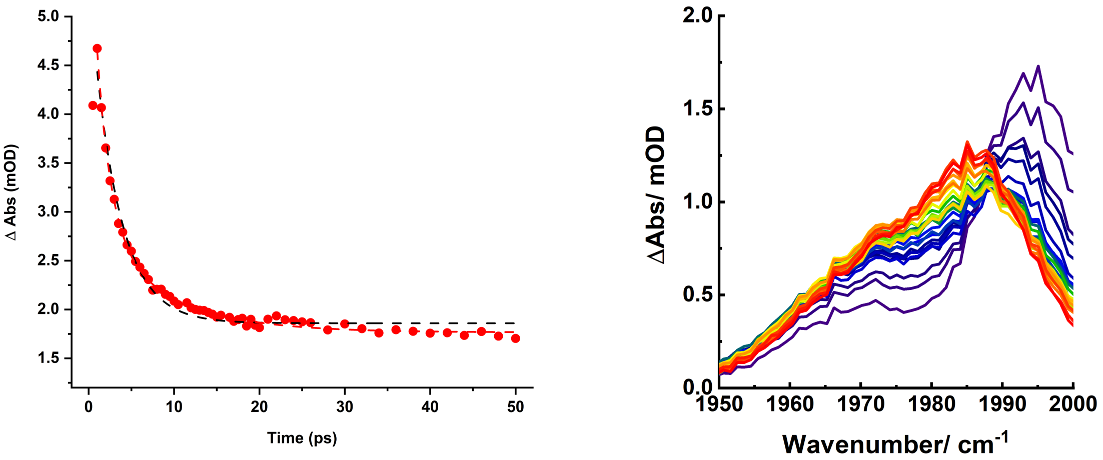

Figure S8. Approximately 5 mg of [$Mn_2(CO)_{10}$] per 10 mL of CH_2Cl_2 solvent pumped with a 400 nm excitation wavelength following the general procedure for time-resolved multiple probe spectroscopy. Left: Kinetics for the loss of the initial photoproduct. The black dashed line is a fit to a monoexponential function, the red dashed line a fit to a biexponential function. Right: Spectra over the first 20 ps with purple, blue, green, yellow, orange, and red progressing from t = 0 ps to t = 20 ps.

Table S1 Summary of data obtained from the kinetic fitting for the band assigned to [$Mn(CO)_5$]* [a] Attempts to fit the acetone data to a biexponential function returned two identical time constants of 2.8 ps. [b] the error for the least mean squares fitting procedure was less than the time resolution of the experiment. Therefore, the latter was used as an estimate of the precision.

	Monoexponential Fit		Biexponential Fit				
Solvent	τ / ps	R^2	τ_1 / ps	τ_2 / ps	$A_1 / 10^{-3}$	$A_2 / 10^{-3}$	R^2
Heptane	5.5 ± 0.8	0.940	1.9 ± 0.6	17 ± 9	1.55 ± 0.26	0.76 ± 0.15	0.974
Toluene	3.4 ± 0.9	0.930	2.2 ± 0.7	191[c]	10.12 ± 5.63	2.53 ± 3.03	0.971
NCMe	1.6 ± 0.5[b]	0.904	0.6 ± 1.3	2.3± 1.8	1.02 ± 1.92	0.52 ± 0.83	0.906
DMSO	1.8 ± 1.0[b]	0.900	0.8 ± 1.0[b]	16.8 ± 8.4	8.72 ± 2.37	1.22 ± 0.19	0.985
Acetone[a]	2.8 ± 1.0[b]	0.970					
THF	3.3 ± 0.5[b]	0.977	0.9 ± 0.5[b]	5.3 ± 0.7	3.52 ± 0.80	1.87± 0.29	0.994
CH_2Cl_2	3.2 ± 0.5[b]	0.981	1.6 ± 0.5[b]	8.2 ± 1.6	3.45 ± 0.23	1.14± 0.25	0.997

The data for the loss in intensity of the peak assigned to [$Mn(CO)_5$]* were fitted to both mono- and biexponential functions and the resulting data are shown in Table S1. Care must be taken when interpreting these data as the intensity and kinetics may be affected by the presence of the overlapping peak from [$Mn(CO)_5$]. However, none of these time constants correlate with changes to the viscosity of the solvent (compare heptane and DMSO for example) which would indicate that diffusion from the solvent cage was occurring. It is proposed that these lifetimes represent energy dissipation from [$Mn(CO)_5$]*.

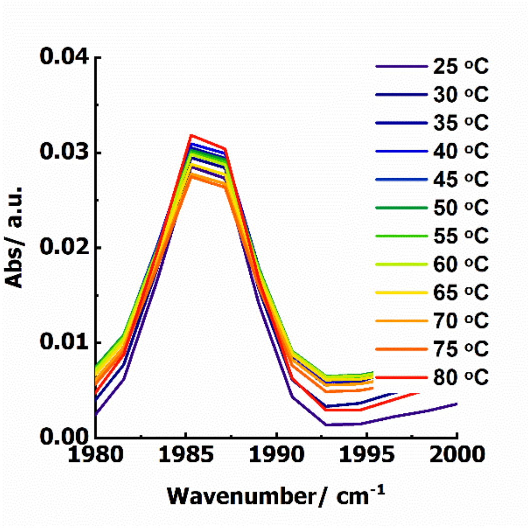

Figure S9. 10 mg of [$Mn_2(CO)_{10}$] dissolved in 10 mL of anhydrous heptane solvent and spectra recorded every 30 s on a Metler Toledo ReactIR ic10 with a K6 conduit SiComp (silicon) probe and MCT detector at varied temperatures measured in situ *with a thermocouple.*

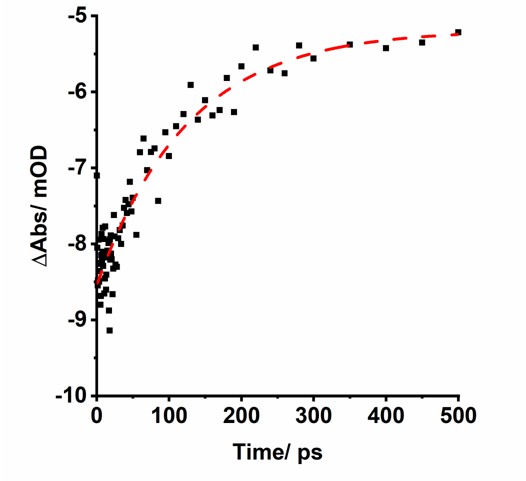

Figure S10. Approximately 5 mg of [$Mn_2(CO)_{10}$] per 10 mL of n-heptane solvent pumped with a 400 nm excitation wavelength following the general procedure for time-resolved multiple probe spectroscopy. Ground state $Mn_2(CO)_{10}$ bleach recovery measured to be 84 ± 3 ps.

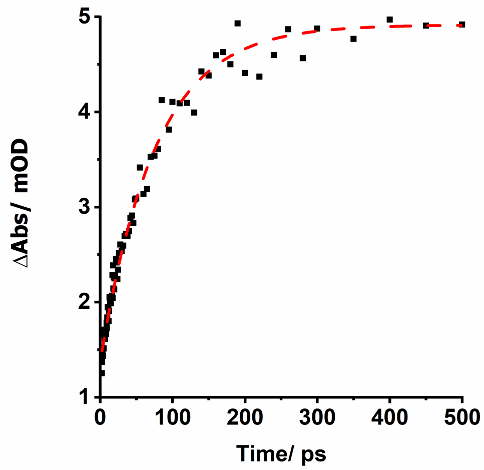

Figure S11. Approximately 5 mg of [$Mn_2(CO)_{10}$] per 10 mL of n-heptane solvent pumped with a 400 nm excitation wavelength following the general procedure for time-resolved multiple probe spectroscopy. Vibrational relaxation of [$Mn(CO)_5$] measured to be 76 ± 8 ps.

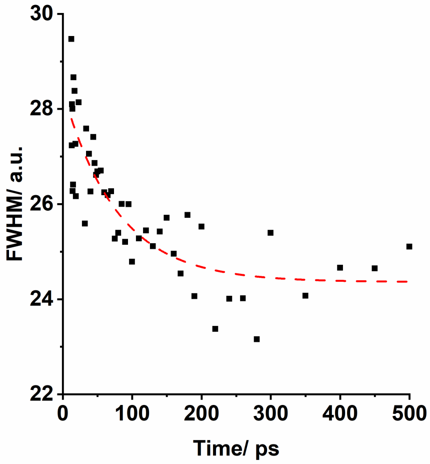

Figure S12. Approximately 5 mg of [$Mn_2(CO)_{10}$] per 10 mL of n-heptane solvent pumped with a 400 nm excitation wavelength following the general procedure for time-resolved multiple probe spectroscopy. Vibrational relaxation using the FWHM of [$Mn(CO)_5$] measured to be 79 ± 39 ps.

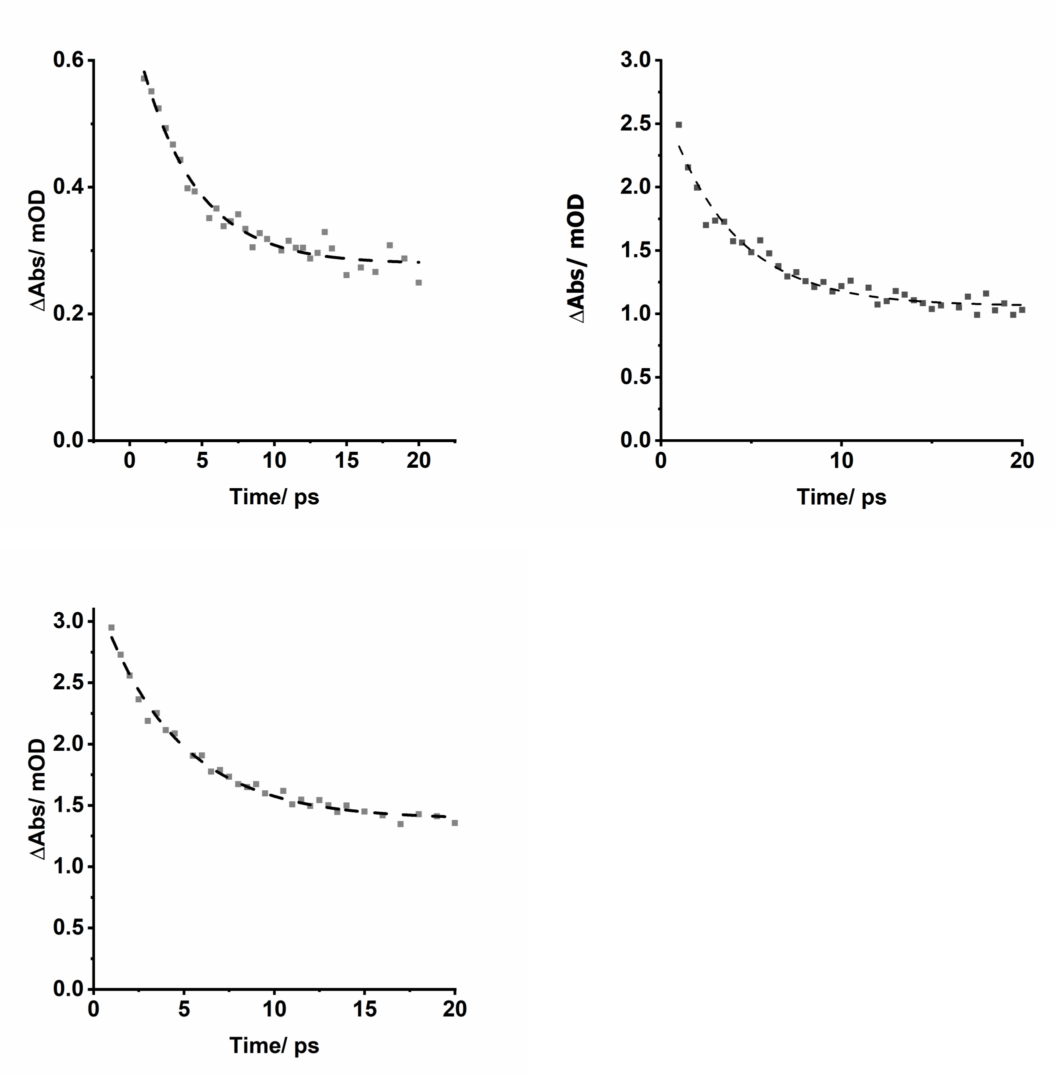

Figure S13. Approximately 5 mg of [$Mn_2(CO)_{10}$] per 10 mL of n-heptane solvent pumped with a 400 nm excitation wavelength following the general procedure for time-resolved multiple probe spectroscopy. Different pump laser powers in n-heptane (125, 250, 500 nJ, top left, top right, and bottom left respectively). Fitted to monoexponential functions.

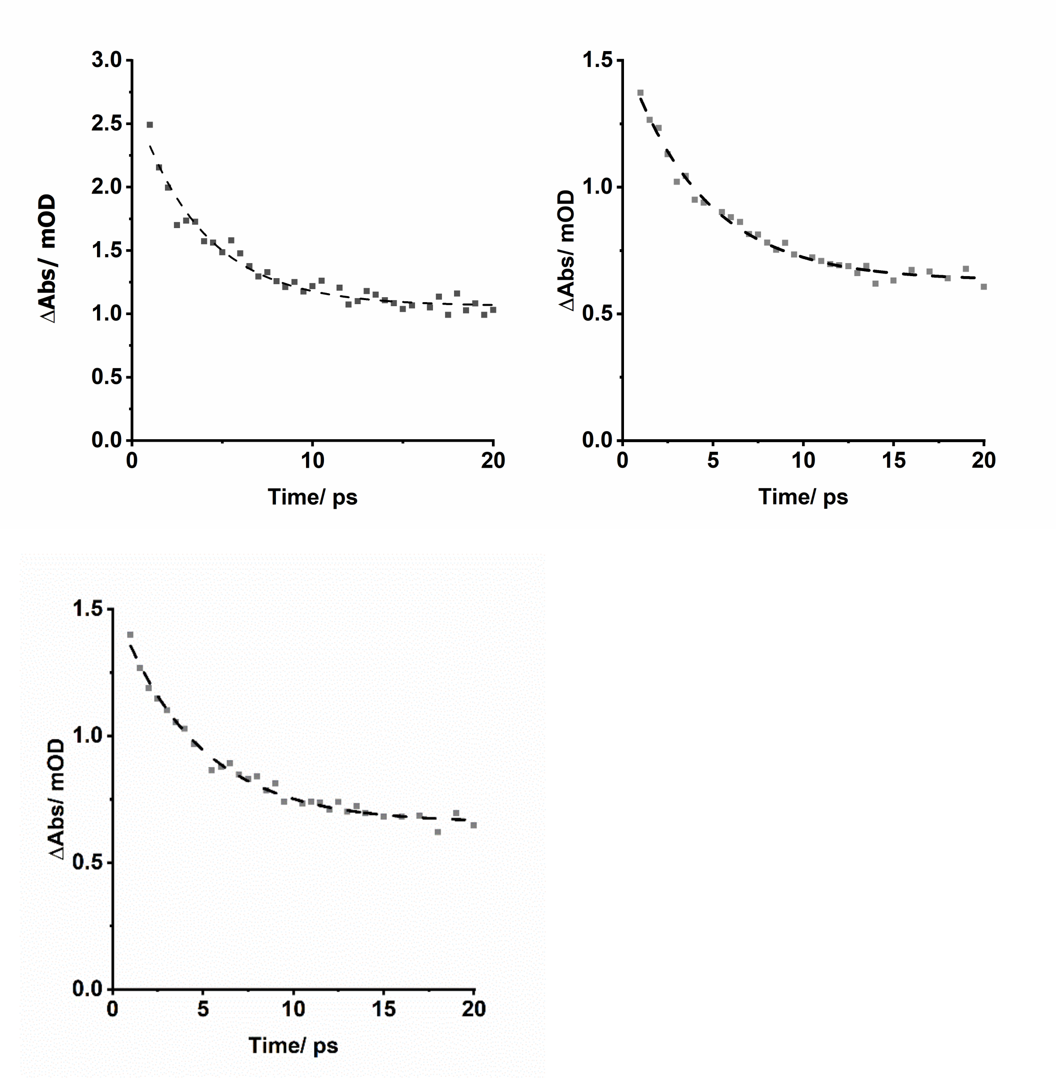

Figure S14. Approximately 5 mg of [$Mn_2(CO)_{10}$] per 10 mL of n-heptane solvent pumped with a 400 nm excitation wavelength following the general procedure for time-resolved multiple probe spectroscopy. Original batch, batch 2, and batch 3 of $Mn_2(CO)_{10}$ top left, top right, and bottom left respectively. Fitted to monoexponential functions.

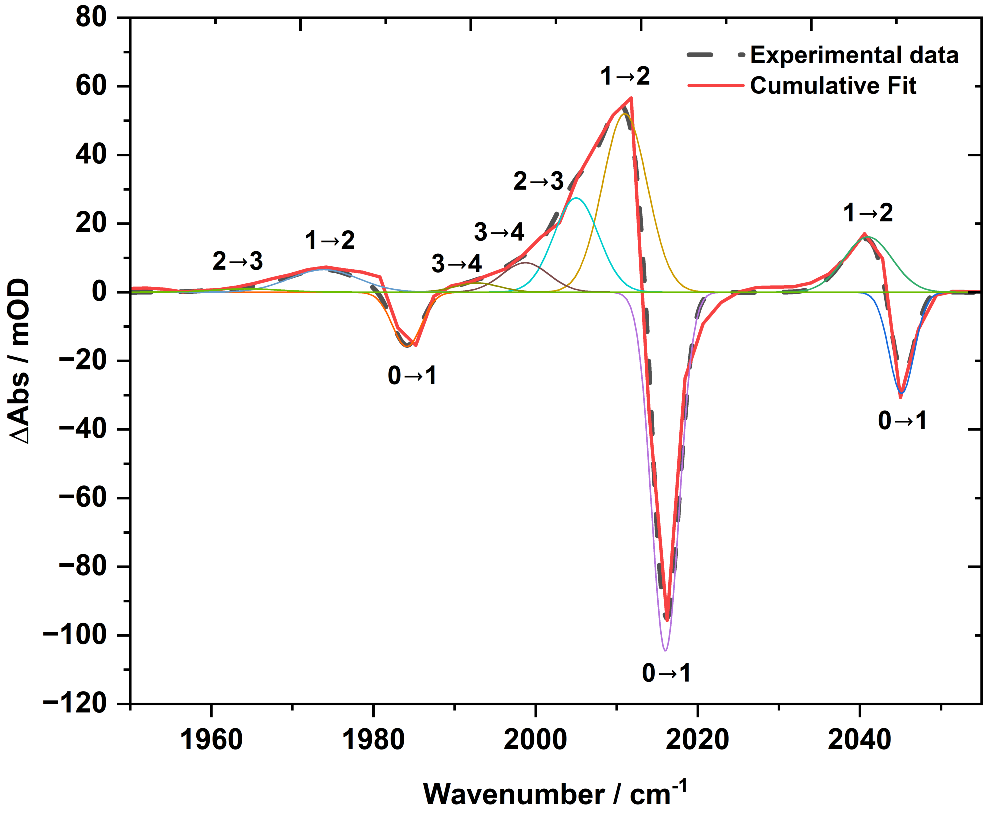

Figure S15.Fitting of the IR-pump IR-probe data for a sample of [$Mn_2(CO)_{10}$] in heptane with T_w = 1 ps. The cumulated fitted data are shown as a red line and the experimental spectrum as a black dashed line. The parameters for the fitted Gaussian peaks are given below.

Index	Assignment	Peak Position / cm^{-1}	Max Height / mOD	FWHM / cm^{-1}
1	0 → 1	2045	-29	4
2	1 → 2	2041	16	7
3	0 → 1	2016	-105	4
4	1 → 2	2011	52	7
5	2 → 3	2005	27	7
6	3 → 4	1999	9	7
7	4 → 5	1993	3	7
8	0 → 1	1984	-16	4
9	1 → 2	1974	7	10
10	2 → 3	1964	1	10

4. Kinetic Modelling

The behaviour of the manganese complexes on a ns-ms timescale was modelled based on the mechanistic pathway shown in Figure S16.

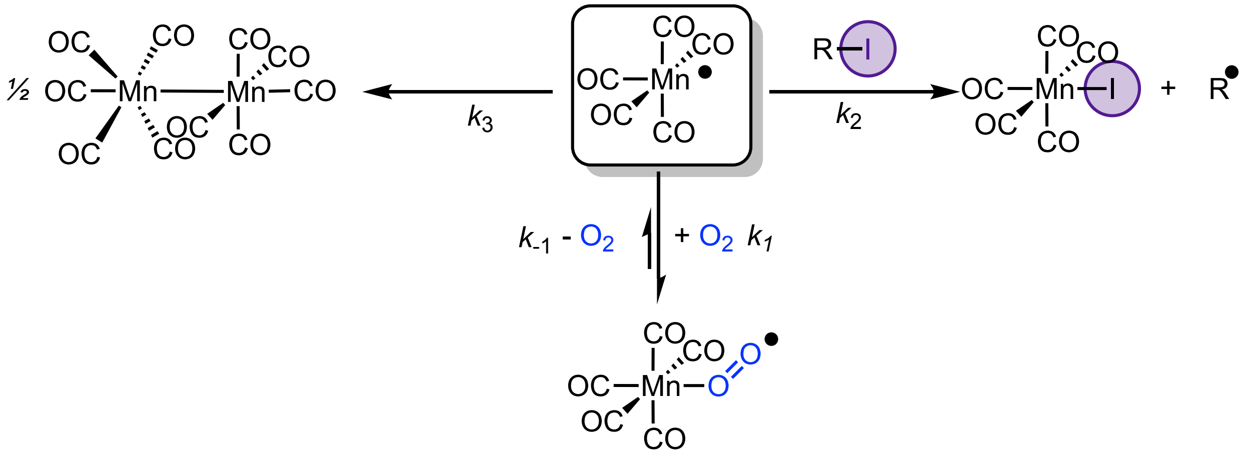

Figure S16 Mechanistic pathways for the fate of [Mn(CO)$_5$] formed from excitation of [Mn$_2$(CO)$_{10}$] at 400 nm.

4.1. Determination of k_1.

Rate constant k_1 was determined by modelling the kinetics for the direct conversion of [Mn(CO)]$_5$ into [Mn(O$_2$)(CO)$_5$] under an atmosphere of air (Figure S21) and O_2 (Figure S22). The later experiment was performed in the specialised apparatus described in Section 5. Under air, the loss of [Mn(CO)]$_5$ obeyed first order kinetics, with a pseudo first order rate constant of $(7.83 \pm 1.39) \times 10^6$ s^{-1}: this increased be a factor of *ca*. four under an atmosphere of O_2, $(2.04 \pm 1.68) \times 10^7$ s^{-1}. Based on the solubility of O_2 in hydrocarbon solvents,[5] under air the [O$_2$] in cyclohexane is 2.38×10^{-3} mol dm^{-3} therefore the second order rate constant k_1 is $(3.29 \pm 0.58) \times 10^9$ mol^{-1} dm^3 s^{-1}.

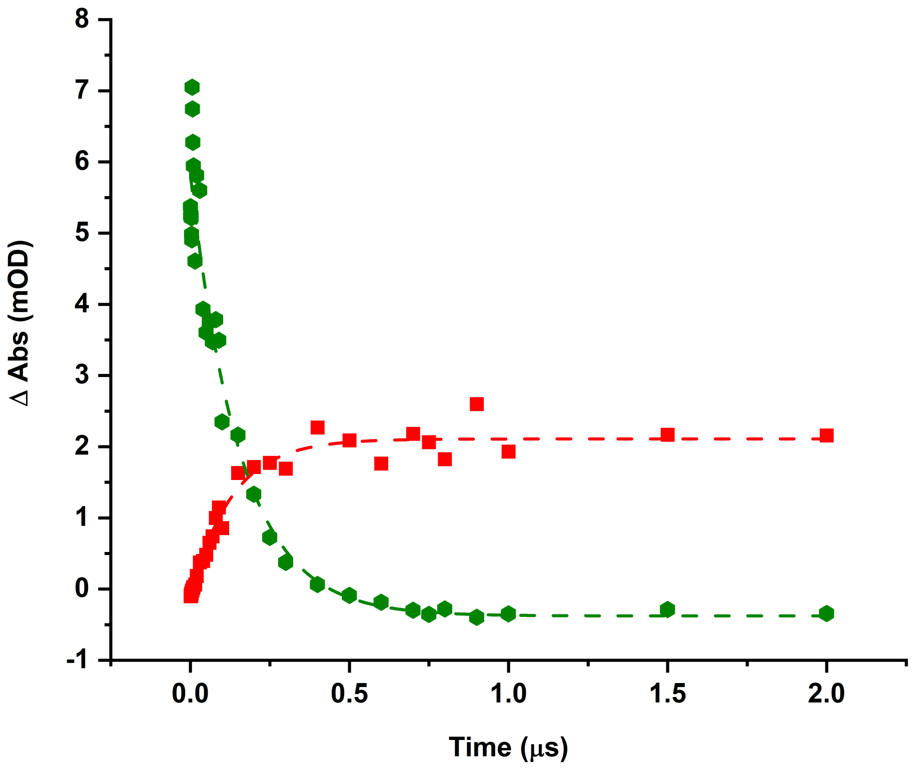

Figure S17. Kinetic data showing the loss of [Mn(CO)$_5$] (green hexagons) and formation of [Mn(O$_2$)(CO)$_5$] (red squares) in a decane solution under an atmosphere of air. The green and red dashed lines are monoexponential fits to the data with rate constants of (6.37 ± 1.52) × 10^6 s^{-1} (R^2 = 0.96) and (7.83 ± 1.39) ×10^6 s^{-1} (R^2 = 0.97) respectively.

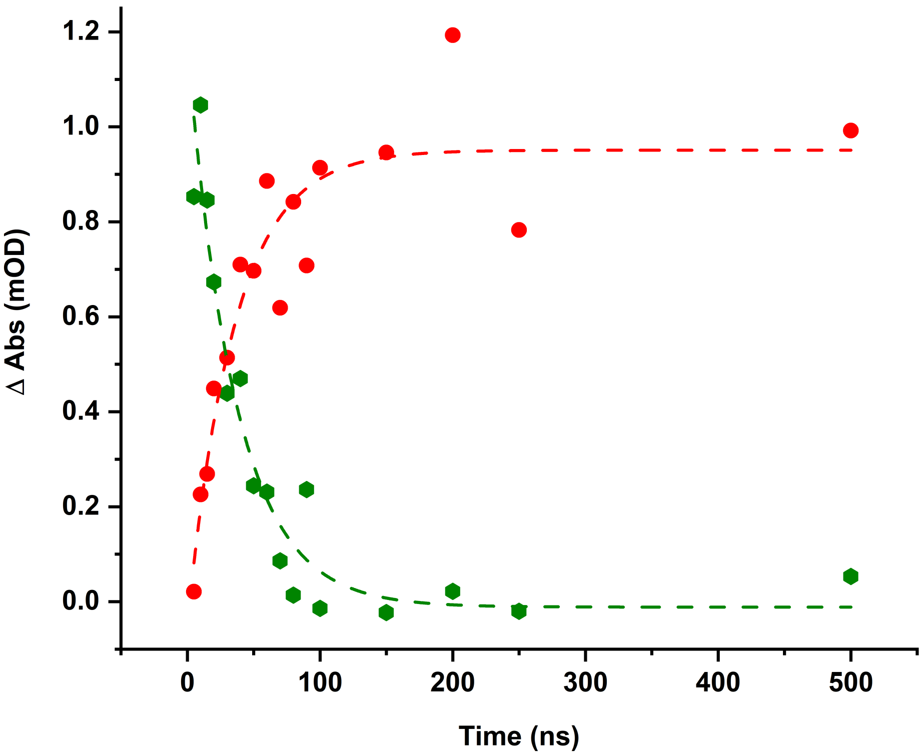

Figure S18. Kinetic data showing the loss of [$Mn(CO)_5$] (green hexagons) and formation of [$Mn(O_2)(CO)_5$] (red squares) in a decane solution under an atmosphere of oxygen. The green and red dashed lines are monoexponential fits to the data with rate constants of (2.75 ± 1.06) × 10^7 s^{-1} (R^2 = 0.93) and (2.80 ± 1.58) ×10^7 s^{-1} (R^2 = 0.85) respectively. This experiment was performed in the apparatus shown in Section 5.

4.2. Determination of k_2 and k_3.

The dimerization of $[Mn(CO)_5]$ to form $[Mn_2(CO)_{10}]$ will be second order in $[Mn(CO)_5]$, whereas the iodide abstraction process will be first order on $[Mn(CO)_5]$ and **1**. Therefore both k_2 and k_3 will be second order rate constants and for the dimerization of $[Mn(CO)_5]$, the pseudo first order approximation does not apply. Therefore, the spectroscopic data were converted from absorbance units to absolute concentration and then baseline corrected. To achieve this, the molar absorption coefficients of $[Mn_2(CO)_{10}]$ were measured ($\varepsilon_{(2045)}$ 1.4 mol^{-1} dm^3 μm^{-1}, $\varepsilon_{(2013)}$ 5.2 mol^{-1} dm^3 μm^{-1}, $\varepsilon_{(1984)}$ 0.8 mol^{-1} dm^3 μm^{-1})and the intensity of the bleach peaks in the difference spectra used to determine the concentration of $[Mn_2(CO)_{10}]$ consumed on photolysis. It was assumed that this was all converted to $[Mn(CO)_5]$ so that concentration versus time data could be generated.

Experiments were performed in which $[Mn_2(CO)_{10}]$ was irradiated at different concentration of **1** under an atmosphere of argon to eliminate the formation of $[Mn(O_2)(CO)_5]$. Two data sets at different concentrations of **1** were identified in which this was successfully achieved. Using the COPASI program,[6] the resulting data were then fitted to a kinetic model in which $[Mn(CO)_5]$ had two competing fates, either dimerization to form $[Mn_2(CO)_{10}]$ or iodide abstraction to generate $[MnI(CO)_5]$. The resulting rate constants are presented in Table S2 and the simulated and actual data in Figure S19 and S20.

Table S2 results from COPASI to determine k_2 and k_3.

Entry	[**1**] / mol dm^{-3}	k_2 / mol^{-1} dm^3 s^{-1}	k_3 / mol^{-1} dm^3 s^{-1}
1	0.0149	(1.47 ± 0.01) × 10^5	(1.108 ± 0.001) × 10^9
2	0.0745	(2.03 ± 0.01) ×10^5	(1.023 ± 0.001) × 10^9
Mean rate constants		(1.75 ± 0.01) × 10^5	(1.065 ± 0.001) × 10^9

The errors in the above rate constants are based on the standard deviations from the fitted data in COPASI. However, given the approximations in the approach and that fact that two values were averaged, the quoted values in the manuscript had a lower degree of precision with k_2 = (1.75 ± 0.30) × 10^5 mol^{-1} dm^3 s^{-1}and k_3 (1.07 ± 0.10) × 10^9 mol^{-1} dm^3 s^{-1}.

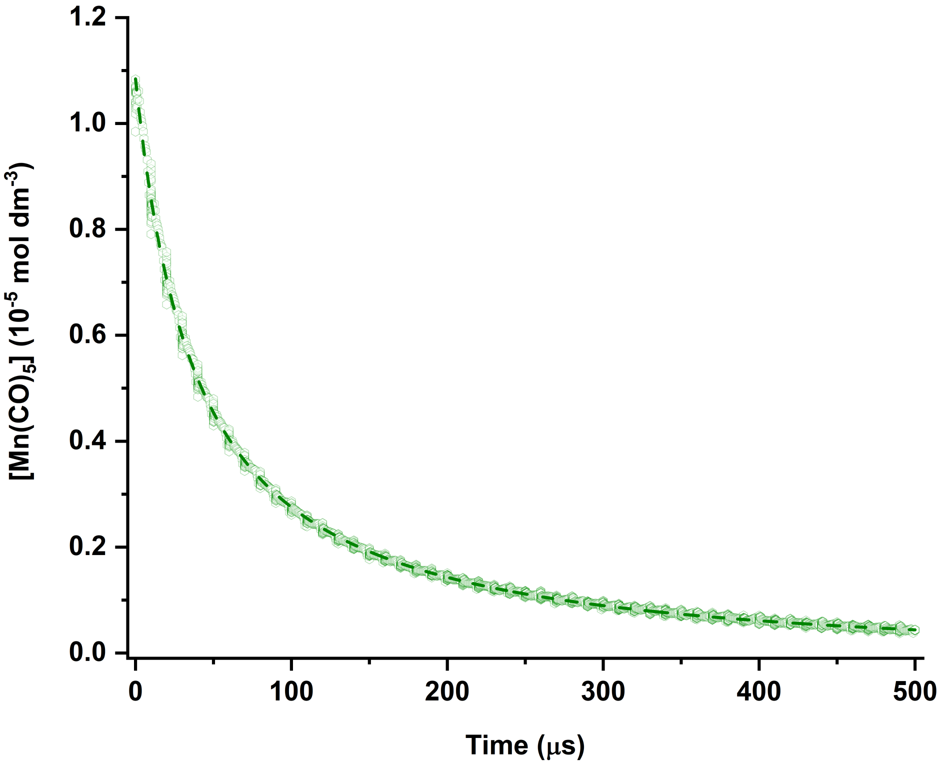

*Figure S19. Kinetic data showing the loss of [Mn(CO)$_5$] (open green hexagons) generated by 400 nm excitation of a cyclohexane solution of 6 mg [Mn$_2$(CO)$_{10}$] containing **1** at a concentration of 0.149 ml dm^{-3} under an argon atmosphere. The green dashed line is a fit to a model in which [Mn(CO)$_5$]$_0$ = (1.084 ± 0.001) × 10^{-5} mol dm^{-3}, k_2* = (1.47 ± 0.01) × 10^5 mol^{-1} dm^3 s^{-1} and k_3 = (1.108 ± 0.001) × 10^9 mol^{-1} dm^3 s^{-1}.

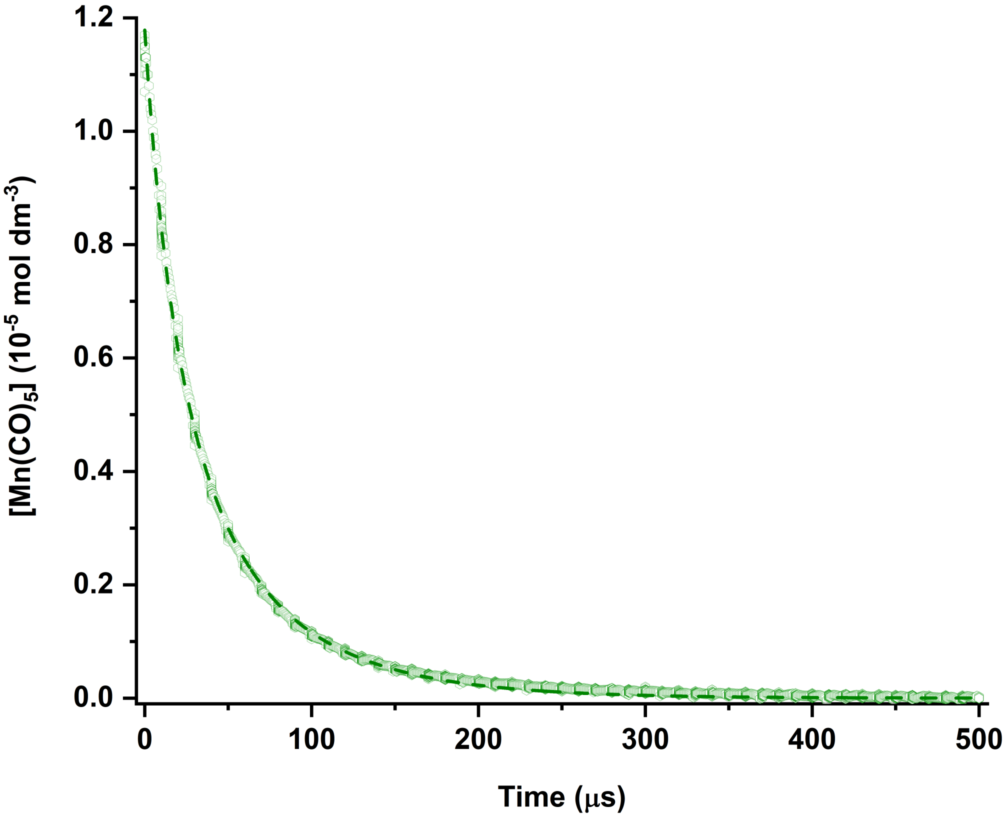

*Figure S20. Kinetic data showing the loss of [Mn(CO)$_5$] (open green hexagons) generated by 400 nm excitation of a cyclohexane solution of [Mn$_2$(CO)$_{10}$] containing **1** at a concentration of 0.745 ml dm^{-3} under an argon atmosphere. The green dashed line is a fit to a model in which [Mn(CO)$_5$]$_0$ = (1.178 ± 0.002) × 10^{-5} mol dm^{-3} k_2* = (2.03 ± 0.01) × 10^5 mol^{-1} dm^3 s^{-1}and k_3 = (1.065 ± 0.001) × 10^9 mol^{-1} dm^3 s^{-1}.

4.3. Determination of k_{-1}.

COPSI modelling was also used to determine the value of k_{-1} using the data from experiments with a pump repetition rate of 50 Hz. In these experiments a cyclohexane solution of $[Mn_2(CO)_{10}]$ and $I(CH_2)_4I$ was excited (400 nm) under an air atmosphere resulting in the initial formation of $[Mn(CO)_5]$, which rapidly (ns timescale) converted to $[Mn(O_2)(CO)_5]$. In this simulation the conversion of $[Mn(O_2)(CO)_5]$ to $[MnI(CO)_5]$ was then modelled with the previously established values of k_1, k_2 and k_3 employed and not allowed to vary. It was assumed that the rate constant for iodide abstraction for **1** and $I(CH_2)_4I$ would be similar. In the simulation the absolute concentration of $I(CH_2)_4I$ was doubled to reflect that the substrate had two potential sites of activation, giving an effective concentration of reactive C–I groups.

The data were then scaled based on an estimate of the manganese concentration generated in these experiments (see Section 4.2). Initial simulations based on the behaviour of $[Mn(O_2)(CO)_5]$ alone indicated that the concentration of $[Mn_2(CO)_{10}]$ generated in these experiments was ca 1%, therefore it was assumed that all $[Mn(O_2)(CO)_5]$ was converted to $[MnI(CO)_5]$ so the concentration of product could then be estimated.

Given the corrections that were needed to the data acquired in this fashion, and the need to approximate the overall concentration of manganese in these experiments, the value of k_{-1} is approximated to 2×10^{-5} s^{-1}.

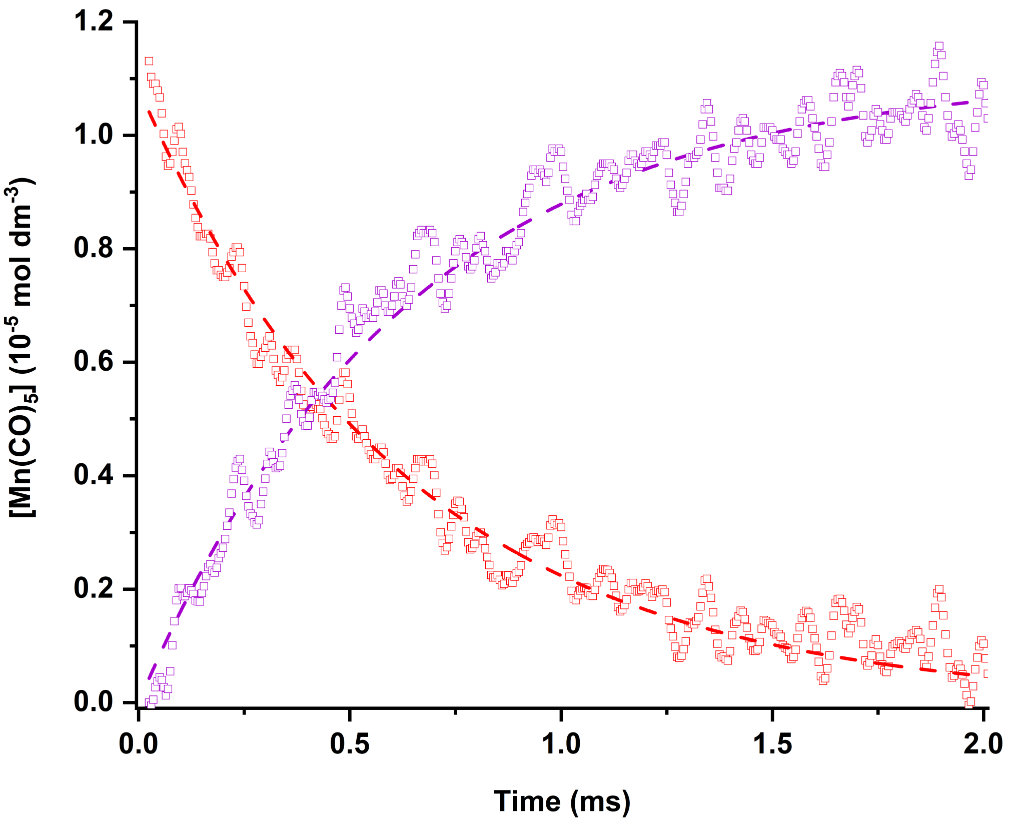

Figure S21. Kinetic data showing the loss [Mn(O)$_2$(CO)$_5$] (red squares) and formation of [MnI(CO)$_5$] (purple squares) in an experiment on a static sample of 6 mg [Mn$_2$(CO)$_{10}$] in 10 ml cyclohexane and 200 μl diiodobutane with pump and probe repetition rates of 20 Hz and 100 kHz respectively. The data have had a 10-point moving average smoothing function applied. The red and purple dashed lines are fits to a model with the fixed parameters, $[I(CH_2)_4I]_{effective}$ = 0.3033 mol dm^{-3}, [O$_2$] = 2.383 × 10^{-3} mol dm^{-3}, k_2 = 1.752 × 10^5 mol^{-1} dm^3 s^{-1} and k_3 = 1.065 × 10^9 mol^{-1} dm^3 s^{-1}, k_1 = 3.282 × 10^9 mol^{-1} dm^3 s^{-1} and variable parameters k_{-1} = (2.40 ± 0.02) × 10^5 s^{-1} and [Mn(O$_2$)(CO)$_5$]$_0$ = (1.131 ± 0.004) ×10^{-5} mol dm^{-3}.

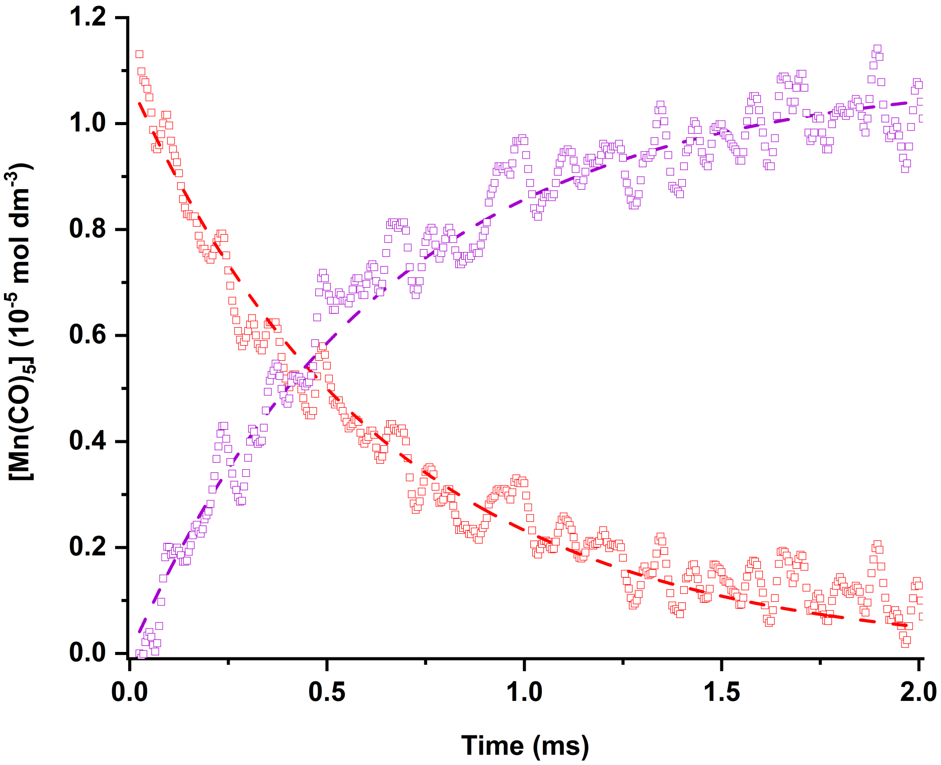

Figure S22. Kinetic data showing the loss $[Mn(O)_2(CO)_5]$ (red squares) and formation of $[MnI(CO)_5]$ (purple squares) in an experiment on a static sample of 6 mg $[Mn_2(CO)_{10}]$ in 10 ml cyclohexane and 400 μl diiodobutane with pump and probe repetition rates of 20 Hz and 100 kHz respectively. The data have had a 10-point moving average smoothing function applied. The red and purple dashed lines are fits to a model with the fixed parameters, $[I(CH_2)_4I]_{effective}$ = 0.3033 mol dm^{-3}, $[O_2]$ = 2.383 × 10^{-3} mol dm^{-3}, k_2 = 1.752 × 10^5 mol^{-1} dm^3 s^{-1} and k_3 = 1.065 × 10^9 mol^{-1} dm^3 s^{-1}, k_1 = 3.282 × 10^9 mol^{-1} dm^3 s^{-1} and variable parameters k_{-1} = (1.16 ± 0.01) × 10^5 s^{-1} and $[Mn(O_2)(CO)_5]_0$ = (1.094 ± 0.004) ×10^{-5} mol dm^{-3}.

5. Procedure for Experiments Performed under Molecular O_2.

Caution mixtures of oxygen gas and hydrocarbons are potentially explosive. Working with oxygen can create higher than normal local oxygen concentrations, which pose a danger to health and must be avoided. Such local concentrations will also increase the flammability of material.

Purging organic solvents with oxygen to create an oxygen-saturated solution increases the risk of fire. The safety data sheets for most flammable solvents with carry the warning "Risk of ignition or formation of inflammable gases or vapours with strong oxidizing agents" in section 10.3.

The use of a peristaltic pump in any flow system to flow oxygen-saturated solutions must be avoided as it can increase this risk of fire as there will be increased local pressure and elevated temperature (albeit a small increase) within the peristaltic pump head.

Liquid flowing through a hose can generate a static charge which further increases the risk of ignition. Build-up of electrostatic charge can also result in small holes in the hose, leading to leaks of fluid or vapour which can ignite. The commonly used hose material in the ULTRA facility is PTFE or PFA, both of which are non-conductive and therefore not able to dissipate static buildup.

Methodology

To mitigate this risk, experiments were performed on a purpose-build stainless steel vessel (See Figures S23 and S24) which was Earthed at all times. The empty apparatus was purged with O_2 gas from a lecture bottle which was connected directly to the experimental vessel through the value "In" for 10 mins. During this purge, the "Vent" was closed, the "Out" valve was open and the Swagelok p cap removed at point (6) so the whole system was purged with O_2. The Swagelok cap at point (6) was then refitted and the "Out" value closed. The sample reservoir was then charged with 30 ml of decane containing 35.1 mg of $[Mn_2(CO)_{10}]$. This solution was then sparged with O_2 with the "Vent" valve open for a minimum of 10 minutes. At this point the attached Harrick cell was filled by the solution by closing the "Vent" gas outlet valve, opening the "Out" valve and removing the Swagelok cap at point (6). The system

was then sealed by placing a Swagelok cap on the outlet to the Harrick cell, all valves were closed and the cylinder was then disconnected from the apparatus.

The whole apparatus was then transferred to the LIFEtime instrument and data acquired. The sample was not flowed during the experiments. To avoid photodegradation of the sample a limited number of pump-probe delays were employed, and the sample was rastered during the acquisition.

Between experimental runs, the reservoir was sparged again with O_2 for 5-10 mins before refilling the Harrick cell.

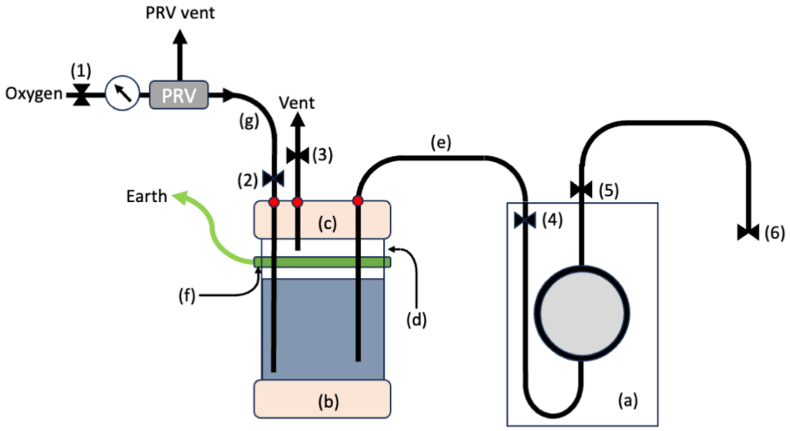

Figure S23 Schematic of "flow" system used to fill a Harrick cell with oxygen saturated solution with stainless-steel tubing throughout (shown in black). (a) Harrick cell with unanodized back plate and taps on inlet and outlet. (b) Swagelok blank cap or welded plug. (c) Swagelok blank cap with welded 1/16 in stainless steel tubes, welds represented by red circles. (d) Wide bore Swagelok stainless-steel tube to form reservoir for sample under investigation. (e) Stainless-steel fill pipe supplying sample to the Harrick cell. (f) Earth cable connected to the stainless-steel sample reservoir with Jubilee clip; the earth cable will be connected to the laboratory earth. (g) Oxygen supply pipe. (h) Glass syringe to draw fluid from the reservoir through the cell during measurement. (1)-(5) 90-degree isolation taps.

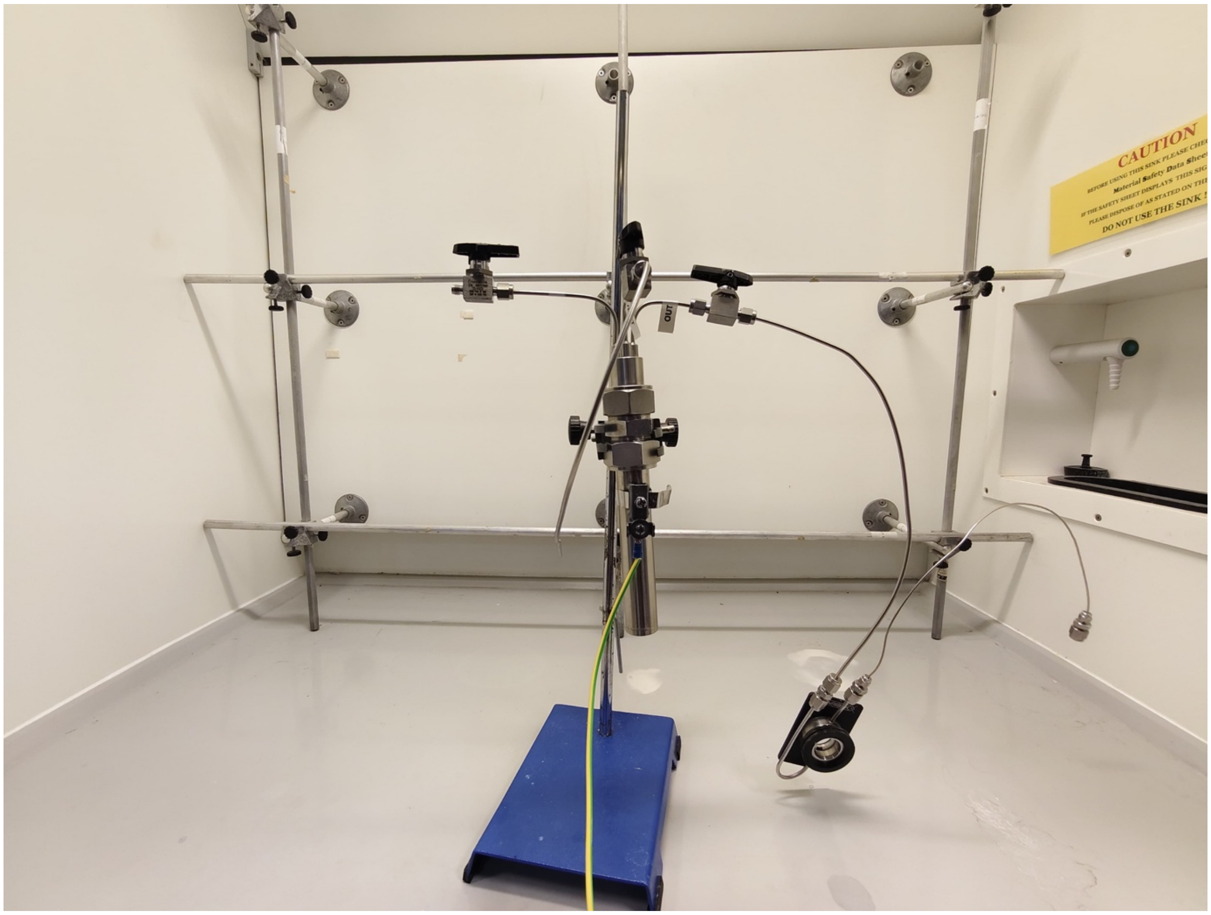

Figure S24. Experimental apparatus used for data acquisition under an atmosphere of O_2.

6. Computational Chemistry

6.1. Methodology

Calculations to determine the relative energies of intermediates were performed using the TURBOMOLE V7.8.1 package using the resolution of identity (RI) approximation.[7-14] Optimisations were performed at the level of theory quoted in Table S3, using a fine *m5* grid followed by frequency calculations at the same level. All minima were confirmed as such by the absence of imaginary frequencies. Energies, xyz coordinates and the vibrational spectra are presented in Section 6.3.

All complete active space self-consistent field (CASSCF) and complete active space second-order perturbation (CASPT2) calculations were carried out in the OPENMOLCAS (v19.11)[15,16] electronic structure package.

The primary CASSCF active space (Figure S21) used to model $Mn_2(CO)_{10}$ comprised fourteen electrons distributed over fourteen orbitals: one of each a_1 and b_2 symmetry, and four of each e_1, e_2, and e_3 symmetry, including the Mn–Mn σ (a_1) and σ* (b_2) orbitals; the degenerate pairs of Mn-centered $3d_{\pi1}$ (e_1), $3\mathrm{d}_{\pi2}$ (e_3), and $3d_{\delta}$ (e_2) orbitals; and six corresponding correlated C=O π* orbitals. Supersymmetry constraints were applied to prevent orbital mixing forbidden under the full D_{4d} symmetry point group but otherwise allowed under the D_2 symmetry point group (the highest symmetry subgroup that is available in OPENMOLCAS). The $1^1\mathrm{A}_1$ (ground), 1^3B_2 (σ* ← σ), 1^3E_1 (σ* ← $3d_{\pi2}$), and 1^1E_1 (σ* ← $3d_{\pi2}$) states were calculated independently within their own spatial and spin symmetry groups, *i.e.*, without state averaging. These calculations are denoted CASSCF(14,14) hereafter, and in the main text.

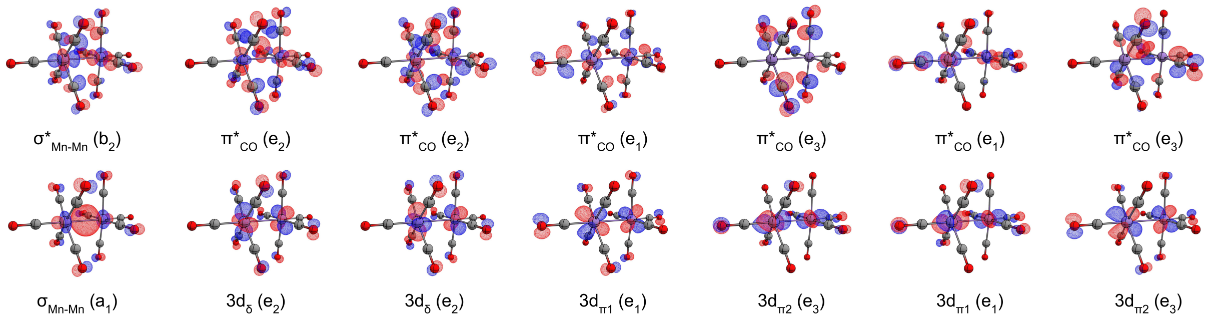

Figure S25. $Mn_2(CO)_{10}$ CASSCF active space, comprising the Mn-Mn σ and σ* orbitals; the degenerate pairs of Mn-centered $3d_{\pi1}$, $3d_{\pi2}$, and $3d_{\delta}$ orbitals; and six corresponding correlated C=O π* orbitals. In total, fourteen electrons were distributed over fourteen orbitals [CASSCF(14,14)].

CASPT2 calculations were carried out on top of the CASSCF(14,14) calculations to validate the results. Ionization potential/electron affinity (IPEA) and imaginary shifts relative to the 0th-order Hamiltonian of 0.25 and 0.8 a.u., respectively, were used.[17] These calculations are denoted CASPT2(14,14) hereafter, and in the main text.

Geometry optimisations of the minima on the 1^1A_1 (ground), 1^3E_1 ($\sigma^* \leftarrow 3d_{\pi2}$), and 1^1E_1 ($\sigma^* \leftarrow 3d_{\pi2}$) state surfaces were carried out at the CASSCF(14,14) level in user-defined internal coordinates so as to enforce conformity to the full D_{4d} symmetry point group.

The ANO-RCC-VDZP[18,19] basis set was used throughout, with scalar relativistic effects accounted for using the Douglas-Kroll-Hess (DKH) Hamiltonian.[20-22]

6.2. Evaluation of the structure and bonding in [Mn(O_2)($CO)_5$]

The structure of [Mn(O_2)($CO)_5$] was optimised with a number of different density functionals and basis sets and the resulting structural metrics, ν(O_2) vibrational modes and spin expectation values compared (Table S3). A similar analysis was then performed for O_2, O_2^- and O_2^{2-}. In summary, at corresponding levels of theory the O–O bond distance in [Mn(O_2)($CO)_5$] is longer than that in O_2 but shorter than in O_2^-, however, ν(O_2) in the complex is very similar to the value of O_2^-. Taken with the orbital analysis reported in Figure 3f, this indicates that the complex is best treated a Mn(I) complex of O_2^-, which is supported by the fact that the ν(CO) bands in the IR spectra of [Mn(O_2)($CO)_5$] and [MnI($CO)_5$] are at similar frequencies (Figures 3d and 3f)

Table S3 Comparison of calculated bond metrics and vibrational spectra for $[Mn(O_2)(CO)_5]$, O_2, O_2^- and O_2^{2-}.

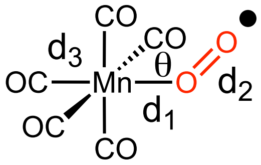

Functional	Basis set	d_1 / Å	d_2 / Å	d_3 / Å	θ / °	νO_2 / cm^{-1}	$\langle S^2 \rangle$
bp86	def2-SVP	2.20408	1.26632	1.82079	120.140	1271	0.812
pbe0	def2-SVP	2.04014	1.27674	1.82455	115.429	1268	0.768
b3lyp	def2-SVP	2.06151	1.29164	1.85127	115.505	1208	0.767
M06-2X	def2-SVP	2.01965	1.29418	1.90334	111.068	1335	0.757
pbe0	def2-TZVPP	2.0730	1.28888	1.82877	115.294	1245	0.762
O_2							
bp86	def2-SVP		1.21612			1596	2.003
pbe0	def2-TZVPP		1.19287			1712	2.009
Expt			1.21			1580	2.000
O_2^-							
bp86	def2-SVP		1.35718			1156	0.751
pbe0	def2-TZVPP		1.32835			1250	0.755
Expt			1.33			1098	0.750
O_2^{2-}							
bp86	def2-SVP		1.62611			634	N/A
pbe0	def2-TZVPP		1.56327			743	N/A
Expt			1.49			877	0

6.3. Predicted vibrational frequencies for key Mn complexes

The MnC≡O vibrational frequencies for the key compounds studies in this manuscript were calculated at both the BP86/SV(P) and PBE0/def2-TZVPP levels of theory. The results from these calculations, and the experimentally determined vibrational modes, are collated in Table S4. The calculated vibrational frequencies are unscaled. Importantly, the calculations demonstrate that at both levels of theory the vibrational modes for both $[MnI(CO)_5]$ and $[Mn(O_2)(CO)_5]$ should be extremely similar, as is observed experimentally. In the calculations, the splitting of the expected *E*-symmetric stretch in $[Mn(O_2)(CO)_5]$ is due to the lowering of the symmetry in the gas phase from C_{4v} induced by the presence of the peroxo-ligand. This may be masked in solution by the width of the observed band (ca. 6 cm^{-1} full width half maximum), although it should be noted that this band also appears to possess a tail to higher energy.

Table S4 Collated experimental and predicted vibrational frequencies for key complexes in this study.

Complex	Experimental frequencies / cm^{-1}	Symmetry	Predicted Frequencies / cm^{-1}			
			BP86/SVP		PBE0/def2-TZVPP	
$[Mn(CO)_5]$	1980	A_1	2020	1.00	2101	1.00
	1987	E	2018	3.93	2104	4.20
$[Mn_2(CO)_{10}]$	1983	B_2	2019	1.00	2098	1.00
	2014	E	2040	6.67	2127	8.32
	2046	B_2	2071	2.10	2138	3.51
$[MnI(CO)_5]$	2004	$A_1^{(1)}$	2035	4.66	2121	4.97
	2045	E	2057	17.83	2154	15.60
	2126	$A_1^{(2)}$	2135	1.00	2224	1.00
$[Mn(O_2)(CO)_5]$	2001	$A_1^{(1)}$	2029	9.75	2120	15.25
	2049	E	2042	20.40	2155	27.31
			2047	18.97	2159	22.22
			2063	1.00	2183	3.88
	2136	$A_1^{(2)}$	2124	1.00	2235	1.00

6.4. Evaluation of the Excited State Manifold of [Mn(CO)$_5$]

The electronic excited manifold of [Mn(CO)$_5$] was explored using CASPT2(14,14) / ANO-RCC-VDZP calculations and the relative energies are shown in Figure S25. This demonstrates that the Franck-Condon state for C_{4v}-symmetric [Mn(CO)$_5$] is unstable with respect to a change in geometry along the D_1 surface (1^2B_1 symmetry). Furthermore, this D_1 state lies at very low energy (3,853 cm^{-1}) and it is proposed is thermally accessed by [Mn(CO)]$_5$ at early times in the 400-pump IR-probe experiments due to the largeexcess thermal energy.

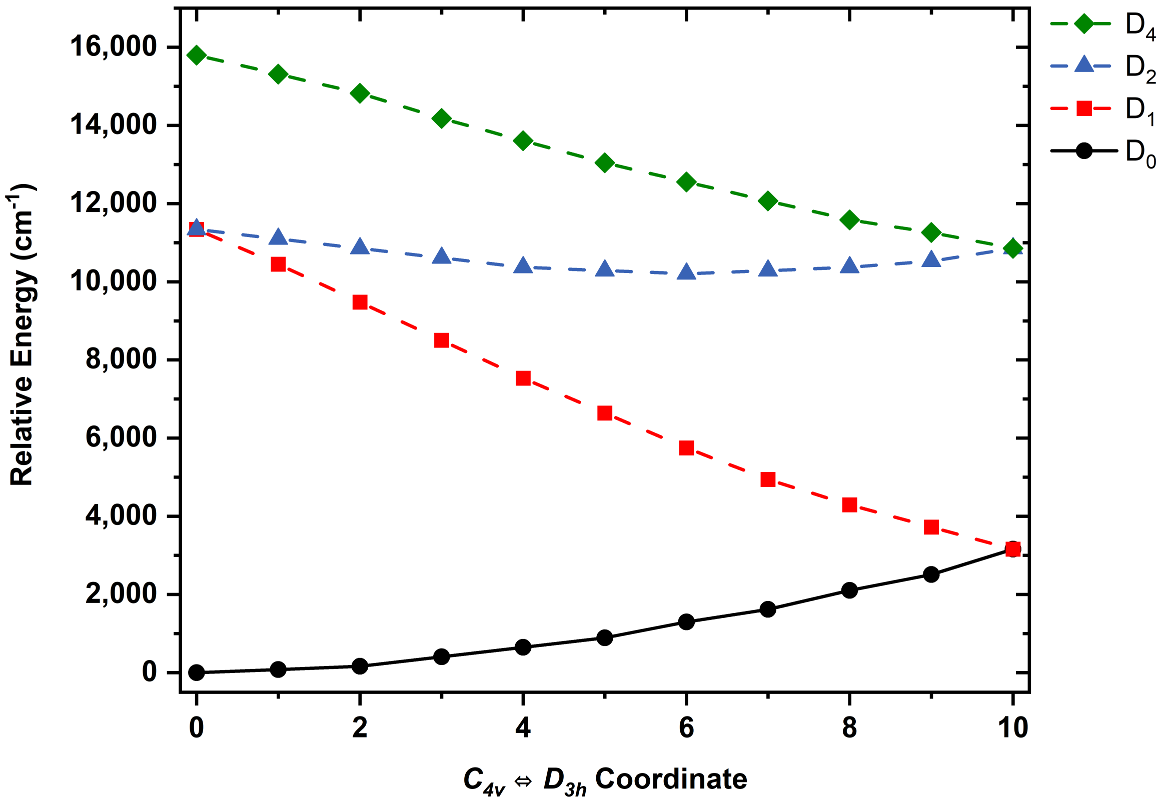

Figure S26. Potential energy surface showing the energies of excited states of [Mn(CO)$_5$] relative to the C_{4v} minimum energy geometry. Energies are at the CASPT2(14,14) / ANO-RCC-VDZP level of theory.

6.5. Collated Energies and xyz coordinates

[Mn(O$_2$)(CO)$_5$] BP86/def2-SV(P)

SCF Energy (au) BP86/SV(P)	-1867.679181369
Zero Point Energy (au)	0.0467734
Chemical Potential (kJ mol^{-1})	8.03

xyz coordinates
13

Mn	-0.1339878	-0.0330559	0.0708233
C	-1.1061415	-0.6990607	1.4588514
C	-0.0608403	1.7185396	0.6905764
C	-1.6634657	0.3825406	-0.8996565
O	-2.6221104	0.6361242	-1.4914733
O	-0.0186714	2.8060441	1.0786162
O	-1.7170282	-1.1274598	2.3473530
O	0.1045634	-2.6342312	-1.4358987
C	1.4828382	-0.3794343	0.9112636
O	2.4941162	-0.5876703	1.4248510
C	0.0154684	-1.6365909	-0.8599352
O	0.9795168	0.7849146	-1.6464472
O	2.2457423	0.7693402	-1.6489241

vibrational spectrum

# mode	symmetry	wave number	IR intensity	selection rules	
#		cm**(-1)	km/mol	IR	RAMAN
1		-0.00	0.00000	-	-
2		0.00	0.00000	-	-
3		0.00	0.00000	-	-
4		0.00	0.00000	-	-
5		0.00	0.00000	-	-
6		0.00	0.00000	-	-
7	a	36.08	0.36825	YES	YES
8	a	57.66	0.00194	YES	YES
9	a	76.58	0.02176	YES	YES
10	a	78.25	0.00010	YES	YES
11	a	95.86	0.03388	YES	YES
12	a	98.21	0.01634	YES	YES
13	a	103.37	0.43358	YES	YES
14	a	104.75	0.00452	YES	YES
15	a	114.80	0.96852	YES	YES
16	a	143.64	0.23564	YES	YES
17	a	180.56	7.01458	YES	YES
18	a	360.24	4.63104	YES	YES
19	a	369.62	0.00032	YES	YES
20	a	402.72	1.37645	YES	YES
21	a	412.62	0.13547	YES	YES
22	a	419.42	0.59708	YES	YES
23	a	430.41	4.92680	YES	YES
24	a	456.45	13.97131	YES	YES
25	a	461.72	14.85900	YES	YES
26	a	473.48	4.40603	YES	YES
27	a	481.09	0.56263	YES	YES
28	a	530.59	0.00327	YES	YES
29	a	541.74	1.07847	YES	YES
30	a	552.32	0.00362	YES	YES
31	a	653.08	122.43674	YES	YES
32	a	658.00	127.42643	YES	YES
33	a	660.69	180.42704	YES	YES

34	a	1271.32	307.68167	YES	YES
35	a	2029.47	643.20242	YES	YES
36	a	2041.98	1345.15141	YES	YES
37	a	2047.16	1250.69908	YES	YES
38	a	2062.85	65.69325	YES	YES
39	a	2124.42	65.94647	YES	YES

$end

[Mn(O_2)($CO)_5$] PBE0/def2-SV(P)

SCF Energy (au) PBE0/def2-SV(P)	-1866.348983756
Zero Point Energy (au)	0.0490751
Chemical Potential (kJ mol^{-1})	16.61

xyz coordinates
13

Mn	-0.1280882	-0.0149580	0.0334365
C	-1.0714841	-0.6883964	1.4425000
C	-0.0613705	1.7320450	0.6720363
C	-1.6765593	0.3861472	-0.9126539
O	-2.6190547	0.6335732	-1.4965502
O	-0.0049502	2.8066852	1.0358813
O	-1.6584948	-1.1109143	2.3264048
O	0.1203500	-2.5995061	-1.4642485
C	1.5192191	-0.3612743	0.8650932
O	2.4914443	-0.5860140	1.4022101
C	0.0167472	-1.6292952	-0.8827294
O	0.9000423	0.7441296	-1.5568206
O	2.1721989	0.6877784	-1.4645598

vibrational spectrum

# mode	symmetry	wave number	IR intensity	selection rules	
#		cm**(-1)	km/mol	IR	RAMAN
1		-0.00	0.00000	-	-
2		0.00	0.00000	-	-
3		0.00	0.00000	-	-
4		0.00	0.00000	-	-
5		0.00	0.00000	-	-
6		0.00	0.00000	-	-
7	a	47.47	0.86483	YES	YES
8	a	64.14	0.04132	YES	YES
9	a	80.81	0.24208	YES	YES
10	a	83.64	0.00465	YES	YES
11	a	102.25	0.00209	YES	YES
12	a	103.41	0.01628	YES	YES
13	a	107.26	0.56030	YES	YES
14	a	113.87	1.07159	YES	YES
15	a	128.54	0.32612	YES	YES
16	a	161.58	0.95193	YES	YES
17	a	214.44	0.47326	YES	YES
18	a	367.69	0.00392	YES	YES
19	a	388.21	1.81935	YES	YES
20	a	395.83	2.78727	YES	YES
21	a	409.54	0.22628	YES	YES
22	a	432.16	0.98058	YES	YES
23	a	441.93	27.28579	YES	YES
24	a	448.69	28.09059	YES	YES
25	a	468.60	2.46690	YES	YES
26	a	483.73	6.75593	YES	YES
27	a	524.08	1.27872	YES	YES
28	a	554.66	0.56462	YES	YES
29	a	561.39	3.28969	YES	YES
30	a	573.98	0.18424	YES	YES
31	a	668.36	155.09139	YES	YES
32	a	669.70	179.67922	YES	YES
33	a	681.69	145.90811	YES	YES
34	a	1267.63	159.35255	YES	YES
35	a	2154.68	783.61505	YES	YES

36	a	2183.30	1434.84097	YES	YES
37	a	2188.59	1133.70148	YES	YES
38	a	2208.67	243.76372	YES	YES
39	a	2260.98	52.00552	YES	YES

$end

[Mn(O_2)($CO)_5$] B3LYP/def2-SV(P)

SCF Energy (au) B3LYP/SV(P)	-1866.955001704
Zero Point Energy (au)	0.0477164
Chemical Potential (kJ mol^{-1})	12.29

xyz coordinates
13

Mn	-0.1296352	-0.0154091	0.0345680
C	-1.0865735	-0.6988532	1.4643829
C	-0.0638223	1.7561753	0.6836791
C	-1.6970110	0.3928982	-0.9279586
O	-2.6385715	0.6431455	-1.5178954
O	-0.0080589	2.8338084	1.0478261
O	-1.6742799	-1.1227945	2.3509232
O	0.1186429	-2.6262049	-1.4771077
C	1.5435664	-0.3664541	0.8778516
O	2.5193485	-0.5909783	1.4145246
C	0.0154322	-1.6538084	-0.8935267
O	0.9068355	0.7520513	-1.5737102
O	2.1941267	0.6964239	-1.4835570

vibrational spectrum

# mode	symmetry	wave number cm**(-1)	IR intensity km/mol	selection rules IR	RAMAN
1		0.00	0.00000	-	-
2		0.00	0.00000	-	-
3		0.00	0.00000	-	-
4		0.00	0.00000	-	-
5		0.00	0.00000	-	-
6		0.00	0.00000	-	-
7	a	47.72	0.84028	YES	YES
8	a	64.23	0.04047	YES	YES
9	a	80.12	0.23482	YES	YES
10	a	83.09	0.00498	YES	YES
11	a	101.08	0.00201	YES	YES
12	a	102.03	0.03479	YES	YES
13	a	105.87	0.61023	YES	YES
14	a	112.22	1.06911	YES	YES
15	a	127.04	0.30518	YES	YES
16	a	159.04	1.03373	YES	YES
17	a	206.78	0.61726	YES	YES
18	a	355.30	0.00693	YES	YES
19	a	369.17	0.35376	YES	YES
20	a	374.65	5.72233	YES	YES
21	a	386.07	0.39152	YES	YES
22	a	415.02	29.05272	YES	YES
23	a	417.26	0.11475	YES	YES
24	a	420.94	33.62105	YES	YES
25	a	442.98	4.61057	YES	YES
26	a	468.20	5.65670	YES	YES
27	a	508.92	1.43228	YES	YES
28	a	539.04	0.22829	YES	YES
29	a	545.81	2.62544	YES	YES
30	a	557.75	0.27223	YES	YES
31	a	640.91	132.66008	YES	YES
32	a	643.98	156.44108	YES	YES
33	a	654.33	125.07244	YES	YES
34	a	1207.55	155.39658	YES	YES
35	a	2116.75	759.00868	YES	YES

36	a	2146.63	1395.01583	YES	YES
37	a	2151.30	1046.30124	YES	YES
38	a	2170.81	287.05959	YES	YES
39	a	2222.51	48.44839	YES	YES

$end

[Mn(O$_2$)(CO)$_5$] M06-2X/def2-SV(P)

SCF Energy (au) M06-2X/def2-SV(P)	-1866.963579604
Zero Point Energy (au)	0.0472746
Chemical Potential (kJ mol^{-1})	7.66

xyz coordinates
13

Mn	-0.1576849	-0.0096615	0.0237845
C	-1.1060029	-0.7220496	1.5123767
C	-0.0771658	1.8192625	0.6996294
C	-1.7653282	0.4127472	-0.9686136
O	-2.6770377	0.6744178	-1.5785723
O	0.0122143	2.8947015	1.0233512
O	-1.6698662	-1.1456298	2.3980220
O	0.1485562	-2.6424945	-1.5423984
C	1.6391373	-0.3527981	0.8536392
O	2.6009673	-0.5772025	1.3946620
C	0.0084704	-1.7048260	-0.9341295
O	0.8846727	0.7329293	-1.5386023
O	2.1590672	0.6206036	-1.3431489

vibrational spectrum

# mode	symmetry	wave number	IR intensity	selection rules	
#		cm**(-1)	km/mol	IR	RAMAN
1		-0.00	0.00000	-	-
2		-0.00	0.00000	-	-
3		-0.00	0.00000	-	-
4		-0.00	0.00000	-	-
5		-0.00	0.00000	-	-
6		0.00	0.00000	-	-
7	a	30.65	1.38500	YES	YES
8	a	60.84	0.06058	YES	YES
9	a	75.05	0.78068	YES	YES
10	a	75.83	0.15329	YES	YES
11	a	89.72	0.02834	YES	YES
12	a	90.26	0.44349	YES	YES
13	a	94.33	0.45790	YES	YES
14	a	99.93	1.99563	YES	YES
15	a	140.25	5.26379	YES	YES
16	a	151.82	2.69264	YES	YES
17	a	270.64	23.79477	YES	YES
18	a	295.80	1.93395	YES	YES
19	a	299.58	0.37996	YES	YES
20	a	307.29	0.45412	YES	YES
21	a	341.43	12.62557	YES	YES
22	a	342.87	49.18136	YES	YES
23	a	355.16	32.75362	YES	YES
24	a	384.48	5.58937	YES	YES
25	a	393.03	1.36422	YES	YES
26	a	449.06	8.94816	YES	YES
27	a	492.36	15.96749	YES	YES
28	a	495.36	3.63208	YES	YES
29	a	503.98	4.46540	YES	YES
30	a	509.35	0.34587	YES	YES
31	a	570.00	100.15127	YES	YES
32	a	578.63	81.10421	YES	YES
33	a	587.82	89.11336	YES	YES
34	a	1335.34	9.17849	YES	YES
35	a	2217.57	820.06086	YES	YES

```
    36         a            2256.32        803.24829        YES      YES
    37         a            2261.91       1222.21690        YES      YES
    38         a            2277.56        357.81230        YES      YES
    39         a            2316.91         22.01272        YES      YES
$end
```

[Mn(O_2)($CO)_5$] PBE0/def2-TZVPP

SCF Energy (au) BP86/SV(P)
Zero Point Energy (au) 0.0485991
Chemical Potential (kJ mol^{-1}) 13.59

xyz coordinates

```
13
Energy = -1867.353292726
Mn   -0.1111065   -0.0027534    0.0081244
C    -1.0577842   -0.6774515    1.4198521
C    -0.0670178    1.7371008    0.6833700
C    -1.6766159    0.3900704   -0.9210277
O    -2.6288267    0.6271703   -1.4829157
O    -0.0286579    2.7978560    1.0721429
O    -1.6449984   -1.0973444    2.2987179
O     0.0980117   -2.6216111   -1.4353097
C     1.5309685   -0.3594416    0.8622486
O     2.4864268   -0.5915900    1.4147153
C     0.0120429   -1.6410244   -0.8797673
O     0.9016719    0.7490236   -1.5662328
O     2.1858854    0.6899955   -1.4739180
```

vibrational spectrum

# mode	symmetry	wave number cm**(-1)	IR intensity km/mol	selection rules IR	selection rules RAMAN
1		-0.00	0.00000	-	-
2		-0.00	0.00000	-	-
3		0.00	0.00000	-	-
4		0.00	0.00000	-	-
5		0.00	0.00000	-	-
6		0.00	0.00000	-	-
7	a	35.99	1.13216	YES	YES
8	a	60.15	0.04181	YES	YES
9	a	77.07	0.24250	YES	YES
10	a	78.65	0.00813	YES	YES
11	a	93.68	0.08739	YES	YES
12	a	94.08	0.07955	YES	YES
13	a	96.06	0.69793	YES	YES
14	a	105.41	1.43471	YES	YES
15	a	121.53	1.10067	YES	YES
16	a	152.35	1.33193	YES	YES
17	a	233.47	2.78657	YES	YES
18	a	370.30	0.00866	YES	YES
19	a	387.52	0.02417	YES	YES
20	a	397.37	5.70062	YES	YES
21	a	405.48	0.61075	YES	YES
22	a	435.98	28.82352	YES	YES
23	a	436.95	0.71854	YES	YES
24	a	439.70	34.30637	YES	YES
25	a	461.95	3.61330	YES	YES
26	a	490.27	0.82790	YES	YES
27	a	532.38	2.96547	YES	YES
28	a	559.41	0.52713	YES	YES
29	a	565.90	0.22395	YES	YES
30	a	567.88	3.42827	YES	YES
31	a	671.87	138.49881	YES	YES
32	a	674.99	148.76642	YES	YES
33	a	688.37	130.13581	YES	YES
34	a	1245.35	104.96212	YES	YES
35	a	2119.80	770.52046	YES	YES

36	a	2155.29	1379.62563	YES	YES
37	a	2159.48	1122.54204	YES	YES
38	a	2182.98	195.81939	YES	YES
39	a	2234.88	50.52258	YES	YES

$end

O_2 BP86/def2-SV(P)

SCF Energy (au) BP86/def2-SV(P)	-150.2260833602
Zero Point Energy (au)	0.0036352
Chemical Potential (kJ mol^{-1})	-41.99

xyz coordinates
2
Energy =

O	0.0000000	0.0000000	0.6080621
O	0.0000000	0.0000000	-0.6080621

vibrational spectrum

# mode	symmetry	wave number	IR intensity	selection rules	
#		cm**(-1)	km/mol	IR	RAMAN
1		0.00	0.00000	-	-
2		0.00	0.00000	-	-
3		0.00	0.00000	-	-
4		0.00	0.00000	-	-
5		0.00	0.00000	-	-
6	a	1595.69	0.00000	YES	YES

$end

O_2 PBE0/def2-TZVPP

SCF Energy (au) PBE0/def2-TZVPP	-150.2356549567
Zero Point Energy (au)	0.0039004
Chemical Potential (kJ mol^{-1})	-41.20

xyz coordinates
2
Energy =

O	0.0000000	0.0000000	0.5964359
O	0.0000000	0.0000000	-0.5964359

vibrational spectrum

# mode	symmetry	wave number	IR intensity	selection rules	
#		cm**(-1)	km/mol	IR	RAMAN
1		-0.00	0.00000	-	-
2		-0.00	0.00000	-	-
3		0.00	0.00000	-	-
4		0.00	0.00000	-	-
5		0.00	0.00000	-	-
6	a	1712.08	0.00000	YES	YES

$end

$[O_2]^-$ BP86/def2-SV(P)

```
SCF Energy (au) BP86/def2-SV(P)            -150.2009418426
Zero Point Energy (au)                     0.0026341
Chemical Potential (kJ mol-1)              -45.17

xyz coordinates
2

O     0.0000000    0.0000000    0.6785915
O     0.0000000    0.0000000   -0.6785915

vibrational spectrum
#  mode     symmetry     wave number   IR intensity    selection rules
#                         cm**(-1)        km/mol          IR     RAMAN
     1                        0.00         0.00000        -        -
     2                        0.00         0.00000        -        -
     3                        0.00         0.00000        -        -
     4                        0.00         0.00000        -        -
     5                        0.00         0.00000        -        -
     6        a            1156.22         0.00000       YES      YES
$end
```

$[O_2]^-$ PBE0/def2-TZVPP

```
SCF Energy (au) PBE0/def2-TZVPP            -150.2334772462
Zero Point Energy (au)                     0.0028482
Chemical Potential (kJ mol-1)              -44.50

xyz coordinates
2

O     0.0000000    0.0000000    0.6641761
O     0.0000000    0.0000000   -0.6641761

vibrational spectrum
#  mode     symmetry     wave number   IR intensity    selection rules
#                         cm**(-1)        km/mol          IR     RAMAN
     1                       -0.00         0.00000        -        -
     2                        0.00         0.00000        -        -
     3                        0.00         0.00000        -        -
     4                        0.00         0.00000        -        -
     5                        0.00         0.00000        -        -
     6        a            1250.22         0.00000       YES      YES
$end
```

$[O_2]^{2-}$ BP86/def2-SV(P)

SCF Energy (au) BP86/def2-SV(P) -149.8181767233
Zero Point Energy (au) 0.0014440
Chemical Potential (kJ mol^{-1}) -49.30

xyz coordinates
2

O	0.0000000	0.0000000	0.8130569
O	0.0000000	0.0000000	-0.8130569

vibrational spectrum

# mode	symmetry	wave number	IR intensity	selection rules	
#		cm**(-1)	km/mol	IR	RAMAN
1		0.00	0.00000	-	-
2		0.00	0.00000	-	-
3		0.00	0.00000	-	-
4		0.00	0.00000	-	-
5		0.00	0.00000	-	-
6	a	633.84	0.00000	YES	YES

$end

$[O_2]^{2-}$ PBE0/def2-TZVPP

SCF Energy (au) PBE0/def2-TZVPP -149.9209946088
Zero Point Energy (au) 0.0016922
Chemical Potential (kJ mol^{-1}) -48.41

xyz coordinates
2

O	0.0000000	0.0000000	0.7816341
O	0.0000000	0.0000000	-0.7816341

vibrational spectrum

# mode	symmetry	wave number	IR intensity	selection rules	
#		cm**(-1)	km/mol	IR	RAMAN
1		0.00	0.00000	-	-
2		0.00	0.00000	-	-
3		0.00	0.00000	-	-
4		0.00	0.00000	-	-
5		0.00	0.00000	-	-
6	a	742.78	0.00000	YES	YES

$end

7. References

[1] Still, W. C.; Kahn, M; Mitra, A. *Rapid chromatographic technique for preparative separations with moderate resolution. J. Org. Chem*. **1978**, *43*, 2923–2925.

[2] Greetham, G. M.; Sole, D.; Clark, I. P.; Parker, A. W.; Pollard, M. R.; Towrie, M. *Time-resolved multiple probe spectroscopy. Rev. Sci. Instrum*. **2012**, *83*, 103107.

[3] Greetham, G. M.; Donaldson, P. M.; Nation, C.; Sazanovich, I. V.; Clark, I. P.; Shaw, D. J.; Parker, A. W.; Towrie, M. *A 100 kHz Time-Resolved Multiple-Probe Femtosecond to Second Infrared Absorption Spectrometer. Appl. Spectrosc.* **2016**, *70*, 645-653.

[4] Monks, B. M. Cook, S. P. *Palladium-Catalyzed Alkyne Insertion/Suzuki Reaction of Alkyl Iodides. J. Am. Chem. Soc.* **2012**, *134*, 15297-15300.

[5] Battino, R.; Rettich, T. R.; Tominaga, T. *The Solubility of Oxygen and Ozone in Liquids. J. Phys. Chem. Ref. Data* **1983**, *12*, 163-178.

[6] Hoops, S.; Sahle S.; Gauges, R.; Lee, C.; Pahle, J.; Simus, N.; Singhal, M.; Xu, L.; Mendes, P.; Kummer, U. *COPASI--a COmplex PAthway Simulator. Bioinform.* **2006**, *22*, 3067-3074.

[7] Császár, P.; Pulay, P. *Geometry optimization by direct inversion in the iterative subspace. J. Mol. Struct.* **1984**, *114*, 31-34.

[8] Ahlrichs, R.; Bär, M.; Häser, M.; Horn, H.; Kölmel, C. *Electronic structure calculations on workstation computers: The program system turbomole. Chem. Phys. Lett.* **1989**, *162*, 165-169.

[9] Deglmann, P.; Furche, F.; Ahlrichs, R. *An efficient implementation of second analytical derivatives for density functional methods. Chem. Phys. Lett.* **2002**, *362*, 511-518.

[10] Deglmann, P.; May, K.; Furche, F.; Ahlrichs, R. *Nuclear second analytical derivative calculations using auxiliary basis set expansions. Chem. Phys. Lett.* **2004**, *384*, 103-107.

[11] Eichkorn, K.; Treutler, O.; Öhm, H.; Häser, M.; Ahlrichs, R. *Auxiliary basis sets to approximate Coulomb potentials. Chem. Phys. Lett.* **1995**, *242*, 652-660.

[12] Eichkorn, K.; Weigend, F.; Treutler, O.; Ahlrichs, R. *Auxiliary basis sets for main row atoms and transition metals and their use to approximate Coulomb potentials. Theor. Chem. Acc.* **1997**, *97*, 119-124.

[13] Treutler, O.;Ahlrichs, R. *Efficient molecular numerical integration schemes. J. Chem. Phys.* **1995**, *102*, 346-354.

[14] von Arnim, M; Ahlrichs, R. *Geometry optimization in generalized natural internal coordinates. J. Chem. Phys.* **1999***, 111*, 9183-9190.

[15] Galván, I. F.; Vacher, M.; Alavi, A.; Angeli, C.; Aquilante, F.; Autschbach, J.; Bao, J. J.; Bokarev, S. I.; Bogdanov, N. A.; Carlson, R. K.; Chibotaru, L. F.; Creutzberg, J.; Dattani, N.; Delcey, M. G.; Dong, S. J. S.; Dreuw, A.; Freitag, L.; Frutos, L. M.; Gagliardi, L.; Gendron, F.; Giussani, A.; González, L.; Grell, G.; Guo, M. Y.; Hoyer,

C. E.; Johansson, M.; Keller, S.; Knecht, S.; Kovacevic, G.; Källman, E.; Li Manni, G.; Lundberg, M.; Ma, Y. J.; Mai, S.; Malhado, J. P.; Malmqvist, P. Å.; Marquetand, P.; Mewes, S. A.; Norell, J.; Olivucci, M.; Oppel, M.; Phung, Q. M.; Pierloot, K.; Plasser, F.; Reiher, M.; Sand, A. M.; Schapiro, I.; Sharma, P.; Stein, C. J.; Sorensen, L. K.; Truhlar, D. G.; Ugandi, M.; Ungur, L.; Valentini, A.; Vancoillie, S.; Veryazov, V.; Weser, O.; Wesolowski, T. A.; Widmark, P. O.; Wouters, S.; Zech, A.; Zobel, J. P.; Lindh, R, *OpenMolcas: From Source Code to Insight*. *J. Chem. Theory Comput*. **2019**, *15*, 5925–5964.

[16] Aquilante, F.; Autschbach, J.; Baiardi, A.; Battaglia, S.; Borin, V. A.; Chibotaru, L. F.; Conti, I.; De Vico, L.; Delcey, M.; Galvan, I. F.; Ferré, N.; Freitag, L.; Garavelli, M.; Gong, X. J.; Knecht, S.; Larsson, E. D.; Lindh, R.; Lundberg, M.; Malmqvist, P. Å.; Nenov, A.; Norell, J.; Odelius, M.; Olivucci, M.; Pedersen, T. B.; Pedraza-González, L.; Phung, Q. M.; Pierloot, K.; Reiher, M.; Schapiro, I.; Segarra-Martí, J.; Segatta, F.; Seijo, L.; Sen, S.; Sergentu, D. C.; Stein, C. J.; Ungur, L.; Vacher, M.; Valentini, A.; Veryazov, V. *Modern quantum chemistry with [Open]Molcas*. *J. Chem. Phys*. **2020**, *152*, 214117.

[17] Ghigo, G.; Roos, B. O.; Malmqvist, P. Å *A modified definition of the zeroth-order Hamiltonian in multiconfigurational perturbation theory (CASPT2)*. *Chem. Phys. Lett.* **2004**, *396*, 142–149.

[18] Roos, B. O.; Lindh, R.; Malmqvist, P. Å.; Veryazov, V. & Widmark, P. O.; Main group atoms and dimers studied with new relativistic ANO basis sets. *J. Phys. Chem. A* **2004**, *108*, 2851–2858.

[19] Roos, B. O.; Lindh, R.; Malmqvist, P. Å.; Veryazov, V.; Widmark, P. O. *New relativistic ANO basis sets for transition metal atoms*. *J. Phys. Chem.* A **2005**, *109*, 6575–6579.

[20] Reiher, M.; Wolf, A. *Exact decoupling of the Dirac Hamiltonian. I. General theory*. *J. Chem. Phys.* **2004**, *121*, 2037–2047.

[21] Reiher, M.; Wolf, A. *Exact decoupling of the Dirac Hamiltonian. II. The generalized Douglas–Kroll–Hess transformation up to arbitrary order*. *J. Chem. Phys.* **2004,** *121*, 10945–10956.

[22] Peng, D. L.; Reiher, M. *An arbitrary order Douglas–Kroll–Hess method with polynomial cost* *J. Chem. Phys.* **2009**, *130*, 044102.

[23] Peng, D. L.; Reiher, M. *Exact decoupling of the relativistic Fock operator*. *Theor. Chem. Acc*. **2012**, *131*, 1081.
